# Efficient and Accurate Epilepsy Seizure Prediction and Detection Based on Multi-Teacher Knowledge Distillation RGF-Model

**DOI:** 10.3390/brainsci16010083

**Published:** 2026-01-09

**Authors:** Wei Cao, Qi Li, Anyuan Zhang, Tianze Wang

**Affiliations:** 1 School of Computer Science and Technology, Changchun University of Science and Technology, Changchun 130022, China; 2 Jilin Provincial International Joint Research Center of Brain Informatics and Intelligence Science, Changchun 130022, China; 3 Zhongshan Institute of Changchun University of Science and Technology, Zhongshan 528437, China

**Keywords:** seizure prediction, seizure detection, Ring-Buffer Gated Recurrent Unit (Ring-GRU), Feature-wise Linear Modulation (FiLM), multi-teacher knowledge distillation, Self-Feedback Prior Mechanism (SFPM)

## Abstract

Background: Epileptic seizures are unpredictable, and while existing deep learning models achieve high accuracy, their deployment on wearable devices is constrained by high computational costs and latency. To address this, this work proposes the RGF-Model, a lightweight network that unifies seizure prediction and detection within a single causal framework. Methods: By integrating Feature-wise Linear Modulation (FiLM) with a Ring-Buffer Gated Recurrent Unit (Ring-GRU), the model achieves adaptive task-specific feature conditioning while strictly enforcing causal consistency for real-time inference. A multi-teacher knowledge distillation strategy is employed to transfer complementary knowledge from complex teacher ensembles to the lightweight student, significantly reducing complexity without sacrificing accuracy. Results: Evaluations on the CHB-MIT and Siena datasets demonstrate that the RGF-Model outperforms state-of-the-art teacher models in terms of efficiency while maintaining comparable accuracy. Specifically, on CHB-MIT, it achieves 99.54% Area Under the Curve (AUC) and 0.01 False Prediction Rate per hour (FPR/h) for prediction, and 98.78% Accuracy (Acc) for detection, with only 0.082 million parameters. Statistical significance was assessed using a random predictor baseline (p < 0.05). Conclusions: The results indicate that the RGF-Model provides a highly efficient solution for real-time wearable epilepsy monitoring.

## 1. Introduction

Epilepsy is a prevalent neurological disorder affecting approximately 50 million people worldwide, with an incidence of about 2 million new cases annually. Seizures are sudden, unpredictable events that severely disrupt daily life and work, making timely and accurate seizure prediction and detection crucial [1]. Electroencephalography (EEG) is a widely used non-invasive technique for these purposes due to its ability to capture real-time neuronal electrical activity and epileptic characteristics [2,3]. However, conventional EEG analysis relies heavily on manual interpretation, which is labor-intensive, subjective, and prone to diagnostic variability, thereby limiting clinical efficiency. Consequently, automated EEG-based approaches have become a major focus in neural engineering research [4,5].

In recent years, deep learning has advanced automated epilepsy monitoring by enabling automatic feature learning and end-to-end processing. Numerous studies report high accuracy on public datasets such as CHB-MIT. For instance, Ji et al. [6] combined a Graph Attention Network (GAT) with a Temporal Convolutional Network (TCN) to capture spatiotemporal dynamics, achieving 99.07% sensitivity (Sen) and a false prediction rate (FPR) of 0.03/h. Wang et al. [7] developed Gatformer, a Transformer with a spatiotemporal attention mechanism, achieving 97.65% Sen and 0.064 FPR/h. Similarly, Zhu et al. [8] proposed a multidimensional Transformer-RNN fusion model (LSTM-GRU) using the Short-Time Fourier Transform (STFT) for time–frequency analysis, achieving 98.24% Sen.

Regarding seizure detection, Abdelhameed and Bayoumi [9] combined a 2D Deep Convolutional Autoencoder (2D-DCAE) with a Bidirectional Long Short-Term Memory network (Bi-LSTM) to extract features from raw EEG, achieving 98.79% accuracy in pediatric epilepsy detection. Liu et al. [10] proposed a hybrid bilinear CNN-LSTM integrating spatial, temporal, and frequency data, achieving recognition accuracies of 98.84% and 98.44%. These studies demonstrate the efficacy of deep learning in analyzing complex EEG data.

Despite their high accuracy, existing deep learning models often suffer from architectural complexity, high parameter counts, and substantial computational costs. These factors hinder clinical deployment, particularly on resource-constrained platforms such as wearable devices [11,12]. Therefore, designing lightweight, deployable models that maintain reliable performance remains a critical challenge.

Lightweight neural network design is primarily achieved through pruning, quantization, and knowledge distillation (KD) [13,14]. KD reduces model size while preserving accuracy by transferring knowledge from a complex “teacher” model to a smaller “student” model without altering the architecture [15,16,17]. For example, Baghersalimi et al. [18] used a KD framework to enable a unimodal student model (using only ECG) to achieve detection accuracy comparable to a multimodal teacher, demonstrating KD’s potential for resource-constrained epilepsy monitoring.

However, most KD studies focus on single tasks, and systematic exploration of unified seizure prediction and detection remains limited [18,19,20]. Since prediction and detection are closely related, independent modeling may reduce efficiency or violate causal consistency—defined as avoiding the use of future information unavailable in real-time [20,21]. Prior work has explored multitask learning and hierarchical distillation [22], but these methods are often overly complex or lack generalizability. Other approaches treat prediction and detection separately [19], increasing system complexity, or use gating mechanisms that fail to strictly ensure causal consistency [23]. Thus, developing a simple, efficient, and causally consistent framework for joint prediction and detection is an urgent priority.

To address these limitations, this study presents the RGF-Model, a lightweight student network for causally consistent seizure prediction and detection. Based on a multi-teacher KD framework integrating Ring-GRU [24] and Feature-wise Linear Modulation (FiLM) [25], the model is jointly guided by separate detection and prediction teachers. FiLM modulates GRU state updates to ensure strict forward-only information flow. Additionally, a Seizure Feature Prediction Module (SFPM) [21] feeds real-time detection states into the prediction branch to enhance sensitivity to preictal signals. The ring structure unifies both tasks, enabling real-time inference with significantly reduced parameters and computational complexity. Experimental results demonstrate that the RGF-Model offers a novel solution for real-time dual-task epilepsy analysis on low-power platforms, achieving performance comparable to or superior to existing complex models. The key contributions of this study are summarized as follows:

1. It introduces the lightweight RGF-Model, achieving unified modeling and real-time inference for prediction and detection through FiLM-modulated GRU gating.
2. It develops a multi-teacher knowledge distillation framework that transfers knowledge from detection and prediction teachers to a lightweight student and incorporates a SFPM to enhance Sen to preictal signals.
3. A ring-structured gating mechanism that enforces causal consistency in temporal tasks was designed, enabling online dual-task operation on resource-constrained devices.
4. It demonstrates on the CHB-MIT and Siena datasets that the model significantly reduces parameter count and computational costs while achieving prediction and detection performance comparable to or better than mainstream methods.

## 2. Materials and Methods

**Figure 1:**
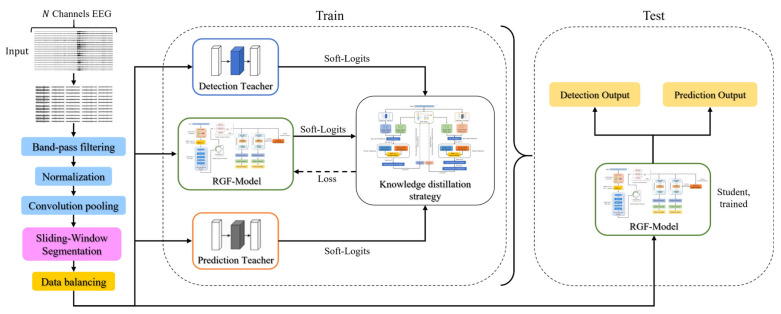
Overall architecture of the model and its workflow.

This chapter describes the experimental datasets and preprocessing procedures, and then details the architectures and workflows of the teacher models and the RGF-Model. The overall framework is shown in Figure 1.

### 2.1. Data Sources

Two representative public EEG datasets were employed to validate the proposed method. The first is the CHB-MIT Scalp EEG database [26], collected in a clinical monitoring unit at Boston Children’s Hospital. To ensure clinical relevance, participants with more than 10 seizures within 24 h were excluded, since seizure prediction is not clinically necessary for patients who experience an average of one seizure every two hours. Consequently, 16 eligible pediatric patients (mean age: 9.5 ± 4.9 years) were retained from the original cohort. Details are provided in Table 1. The second dataset is the Siena Scalp EEG database [27], acquired in a hospital setting at the University of Siena. To align with the CHB-MIT protocol and prevent overfitting caused by insufficient data, patients with fewer than three seizures were excluded. Ultimately, 8 adult patients (mean age: 45.3 ± 14.2 years) met the inclusion criteria [28]. Details are provided in Table 2.

**Figure 2:**
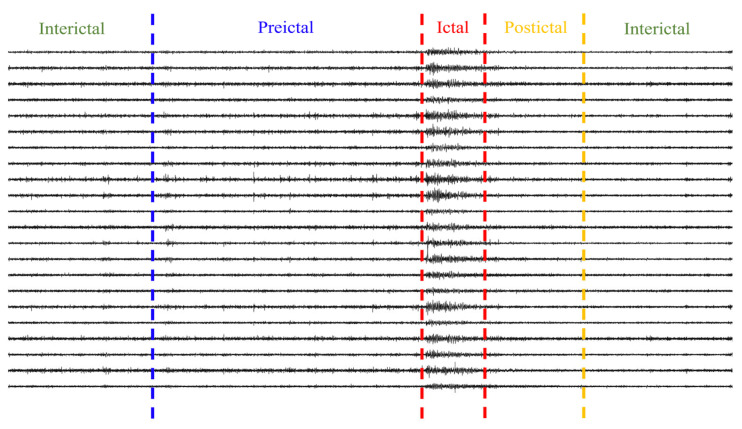
In the seizure prediction task, four states are identified: interictal, preictal, ictal, and postictal. Each of the 22 waveforms represents one of the 22 channels of EEG data.

For the seizure prediction task, most current studies segment recordings into interictal, preictal, ictal, and postictal periods [29,30]. The preictal state denotes the period preceding an impending seizure, as shown in Figure 2. In this study, the preictal segment was treated as the positive class and an interictal segment distant from the seizure as the negative class, thereby formulating the prediction task as a binary classification between interictal and preictal states [31]. For impending seizure prediction, the protocol followed Li et al. [32], which treated consecutive seizures separated by less than 30 min as a single event and defined the onset of the earlier seizure as the event onset.

### 2.2. Data Preprocessing

The preprocessing steps for raw EEG data consisted of signal segmentation, bandpass filtering, normalization, and convolution and pooling operations. EEG signals are susceptible to artifacts from blinking, electromyographic activity (EMG), line noise, and sweating. Therefore, a 0.5 to 40 Hz bandpass filter was applied to the raw multichannel EEG to attenuate high-frequency noise, including power line interference and electromyographic artifacts, while preserving seizure-related frequency components. Subsequently, baseline drift was removed by zero-mean normalization, in which the mean of each channel was subtracted to eliminate slowly varying DC offsets that could affect subsequent analysis. Given prominent interference at 60 Hz in CHB-MIT and 50 Hz in Siena, additional filtering was applied to CHB-MIT in the 57 to 63 Hz and 117 to 123 Hz bands and to Siena in the 49 to 51 Hz and 99 to 101 Hz bands to remove this interference [33,34]. All other bandpass and normalization procedures were kept the same. In addition, any residual DC component was removed.

Next, a unified sliding-window strategy was adopted to partition continuous EEG into fixed-length segments for model training and analysis. A 2-s window with 50 percent overlap was applied in both prediction and detection, because a 2-s window within the 0.5 to 40 Hz band provides approximately 0.5 Hz frequency resolution, which is more robust than a 1-s window. A 50 percent overlap yields an update rate of 1 Hz, smoothing outputs and suppressing transient noise without materially increasing computational load. This strategy aligns with the causal front end and post-processing used in this study, balances prediction lead time and detection latency, and is applicable to datasets with different sampling rates. This configuration has been validated in prior high-quality studies [35,36].

To preserve as much useful signal information as possible, both datasets were retained at their original sampling rates without resampling. Each CHB-MIT window contained 512 samples (256 Hz × 2 s). Each Siena window contained 1024 samples (512 Hz × 2 s). Given the 512 Hz rate for Siena and the 256 Hz rate for CHB-MIT, a rate-aware temporal chunking module was introduced at the front end to partition each 2 s window into a fixed number of tokens without resampling, thereby eliminating computational disparities due to different sampling rates and maintaining comparability [37]. Chunking used a stride of 8 samples for CHB-MIT and 16 samples for Siena, which yielded the same number of tokens per window entering the encoder and enabled efficient cross-dataset processing.

Each window was then assigned a label based on the time period it covered. For seizure detection, if the sliding window overlapped with any ictal segment, it was labeled as seizure as the positive class, and otherwise it was labeled as non-seizure as the negative class. For seizure prediction, the preictal period was defined as the 30 min preceding seizure onset. Windows entirely within this 30-min preictal interval were labeled as the preictal class, whereas those occurring at least 30 min from the onset of any seizure were labeled as the interictal class.

**Figure 3:**
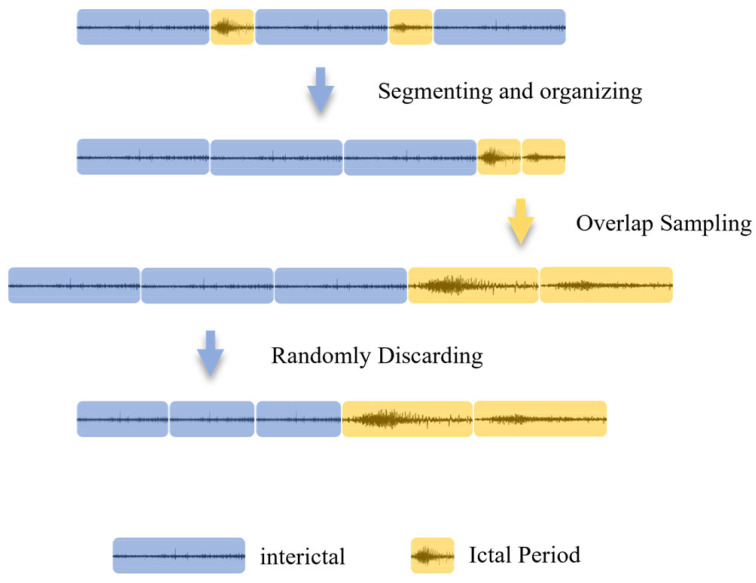
Data processing methods to address data imbalances.

Because seizure events, including the aura phase, occur far less frequently than non-seizure activity over time, the resulting number of windows was severely imbalanced [38]. To mitigate class imbalance, a hybrid sampling strategy was employed after generating the initial sliding-window samples, combining oversampling and undersampling. Specifically, positive-class windows were moderately oversampled, and random downsampling was applied to reduce the volume of interictal data, thereby approximately balancing the number of samples between ictal and interictal periods. This procedure improved the model’s recognition Acc for the minority class. The above balancing procedure was applied only to the training set, and the test set retained its original distribution to more accurately reflect real-world deployment scenarios. The detailed procedure is shown in Figure 3.

### 2.3. Teacher Model

In recent years, deep-learning-based epileptic seizure prediction and detection models have increasingly supplanted traditional approaches and become a major research focus due to their strong feature learning and end-to-end modeling capabilities. To facilitate efficient knowledge transfer and enhance student model performance, eight representative deep learning architectures were chosen as teachers in this study [6,7,8,9,10,39,40,41], covering prediction and detection. The selected models included 3D Convolutional Neural Networks (3D-CNNs), 2D-DCAEs, Long Short-Term Memory networks (LSTMs), GRUs, GATs, self-attention modules, and Transformers, representing mainstream architectures. Together, these teachers extracted deep patterns from spatial, temporal, and frequency domains, thereby enhancing the performance of seizure prediction and detection.

The first teacher model is a 3D-CNN–BiLSTM network with an integrated Convolutional Block Attention Module (CBAM-3D CNN-LSTM) [39]. The model first converts raw temporal EEG into time–frequency representations using the STFT and then extracts local spatiotemporal features with a 3D-CNN. The 3D-CNN performs convolutions jointly across time, frequency, and spatial dimensions to capture multiscale time–frequency patterns in multichannel EEG. Building on this, CBAM adaptively emphasizes channel and spatial features critical for prediction through sequential channel and spatial attention. The channel attention weights are computed as in Equation (1).

(1) $$M_{c}(F)=\sigma (W_{2}\mathrm{RELU}(W_{1}F_{avg})+W_{2}\mathrm{RELU}(W_{1}F_{max})),$$

where $M_{c}(F)$ denotes the channel attention weights, $F_{avg}$ and $F_{max}$ represent the global average and maximum pooling results of the input feature map F. σ corresponds to the Sigmoid function. and $W_{1}$ and $W_{2}$ denote the weight parameters of the two-layer fully connected network used to model nonlinear channel relationships. Through this mechanism, CBAM effectively enhances the model’s focus on critical frequency bands and salient channel features. Finally, the model employs Bi-LSTM to capture the long-range temporal dependencies of EEG signals and to construct complete contextual information through the fusion of forward and backward hidden states, thereby improving the prediction Acc of impending epileptic seizures.

The second seizure prediction teacher adopts an architecture integrating a Graph Attention Network with a Temporal Convolutional Network (GAT-TCN) [6]. The model represents multichannel EEG as a graph, uses GAT to model spatial topology among channel nodes, and computes inter-node attention coefficients to obtain a more comprehensive spatial representation. Subsequently, a TCN captures long-range temporal dependencies using causal convolutions that restrict the receptive field to past information. The convolution operation varies with the dilation factor, as shown in Equation (2).

(2) $$y(n)=\sum_{i=0}^{K-1}w_{i}\cdot x(n-d\cdot i).$$

In Equation (2), $w_{i}$ denotes the convolutional filter kernel coefficient, K represents the kernel length, d indicates the dilation rate, x(n) denotes the input sequence, and y(n) represents the output sequence. The multilevel dilated convolution enables the model’s receptive field to grow exponentially and effectively captures pattern variations across different time scales. By combining the spatial features extracted by GAT with the temporal features learned by TCN, the model more accurately predicts the complex dynamic processes preceding seizures.

The third seizure prediction teacher employs a multidimensional Transformer with a parallel LSTM–GRU fusion architecture [8]. The model first uses a Transformer encoder to capture global relationships across temporal segments and frequency components of the EEG sequence via multi-head self-attention. Multi-head self-attention computes attention in parallel across heads, concatenates the head outputs, and projects them back to a unified representation, as in Equation (3).

(3) $$H=Concat\left(H_{1},H_{2},\ldots ,H_{h}\right)W^{O},$$

where $H_{i}$ denotes the output of the i attention head, and $W^{O}$ represents the output projection matrix used to map the concatenated multi-head results back to the original feature dimension. The global features extracted by the Transformer are then fed into LSTM and GRU networks in parallel. LSTMs excel at capturing long-range dependencies, whereas GRUs are streamlined and computationally efficient. The outputs of the two networks are fused through a gating mechanism to achieve integrated modeling of EEG temporal and frequency features, thereby enhancing seizure prediction performance.

The fourth seizure prediction teacher adopts the GATformer architecture, integrating a Graph Attention Network with a Transformer [7]. The model first uses GAT to extract spatial associations among channels, where each node feature vector is weighted and aggregated with its neighbors to update the representation. The attention coefficients are computed by applying a linear mapping to the concatenated node features, followed by an activation function, as shown in Equation (4).

(4) $$\alpha_{ij}=\frac{\exp(\mathrm{LeakyReLU}(a^{T}[Wh_{i}∥Wh_{j}]))}{\underset{k\in N(i)}{\sum }\exp(\mathrm{LeakyReLU}(a^{T}[Wh_{i}∥Wh_{k}]))},$$

where W denotes the trainable linear transformation matrix, a represents the attention vector, $h_{i}$ and $h_{j}$ are the input features of nodes i and j, respectively, and N(i) denotes the set of neighbors of node i. After the weighted inter-channel dependency representation is computed by the GAT layer, long-range temporal dependencies are further modeled using the Transformer encoder. Its self-attention computation follows the scaled dot-product formulation, as shown in Equation (5).

(5) $$\mathrm{Attention}\left(Q,K,V\right)=\mathrm{softmax}\left(\frac{QK^{T}}{\sqrt{d_{k}}}\right)V,$$

where Q, K, and V denote the query, key, and value matrices, respectively, and $d_{k}$ represents the scaling factor of the key vectors. By deeply integrating the spatial features extracted by GAT with the temporal features learned by the Transformer, GATformer accurately characterizes the complex spatio-temporal coupling patterns of multichannel EEG signals preceding seizures, thereby substantially improving the Sen and Acc of seizure prediction.

For the seizure detection task, the first teacher model employs a two-dimensional deep convolutional autoencoder in conjunction with a bidirectional long short-term memory network (SDCAE) [9]. The model first takes raw multichannel EEG segments (channels by time) as input to the 2D-DCAE, which learns low-dimensional, information-dense representations via unsupervised autoencoding. The convolutional encoder progressively extracts higher-order features through stacked convolution and pooling layers, and the decoder reconstructs the input to preserve fidelity and encourage robust representations. The encoded features are then passed to a bidirectional LSTM classifier, where concatenating forward and backward hidden states allows the model to use both past and future context, enhancing recognition of transient seizure patterns. In this setting, the LSTM hidden-state update is computed as in Equation (6).

(6) $$h_{t}=o_{t}⊙\tan\cdot h(c_{t}),$$

where $h_{t}$ denotes the hidden state of the LSTM at time step t. $o_{t}$ represents the output gate activation, which controls the proportion of the cell state tanh released after the nonlinear transformation $c_{t}$. And ⊙ indicates element-wise multiplication. This formulation reflects the regulation of hidden-state information flow by the output gate, thereby enhancing the model’s ability to discriminate transient features within the sequence.

The second seizure detection teacher model adopts a CNN-LSTM architecture with a self-attention (CNN-LSTM-SAT) mechanism [40]. The model first extracts local features with a 1D-CNN, then an LSTM captures temporal dependencies, and a self-attention module dynamically assigns weights to time steps to emphasize salient segments. The attention weights are computed as in Equation (7).

(7) $$\alpha_{ij}=\mathrm{softmax}\left(\frac{Q_{i}K_{j}^{T}}{\sqrt{d_{k}}}\right),$$

where $\alpha_{ij}$ denotes the attention weight between query vector $Q_{i}$ and key vector $K_{j}$, while $d_{k}$ represents the dimensionality of the key vector, which determines the degree of scaling. Through this mechanism, the model adaptively focuses on seizure-related time periods, thereby enhancing the generalization capability of seizure detection.

The third seizure detection teacher employs a Graph Transformer Network (GTN) architecture [41]. The model represents each EEG channel as a graph node and first uses a GAT module to perform weighted aggregation of multichannel information, with attention weights computed as in Equation (4), to obtain spatial dependencies among brain regions. The graph features are then passed to a Transformer module to encode global temporal relationships, enabling joint modeling of brain-region interactions and seizure-related patterns. This fusion architecture improves seizure detection Acc and enhances adaptability across patients.

The fourth epilepsy detection teacher model adopts a bilinear CNN–LSTM architecture that fuses multidimensional features (CNN-LSTM-Bilinear) [10]. The model first filters discriminative feature components through a single-channel self-attention mechanism. It then employs a convolutional neural network to extract local EEG patterns in the temporal, spatial, and frequency domains, while capturing long-range dependencies across time steps using an LSTM. In the feature fusion stage, the model introduces a bilinear pooling layer to perform second-order interactions between convolutional and temporal features, computed as shown in Equation (8).

(8) $$B=f_{c}\cdot f_{t}^{T},$$

where B denotes the bilinear feature matrix, while $f_{c}$ and $f_{t}^{T}$ represent the output feature vectors of the convolutional network and the LSTM, respectively. Subsequently, Equation (9) is applied to perform sign-sqrt transformation and normalization on B.

(9) $$z=\mathrm{sign}(B)\sqrt{\left|B\right|},\hat{z}={z/\left\|z\right\|}_{2}.$$

Equation (9) stabilizes the distribution of second-order features and improves their discriminative power. Bilinear pooling captures second-order correlations between convolutional and temporal features and thereby enhances the model’s capacity to represent complex relationships.

### 2.4. RGF-Model

To achieve efficient prediction and detection of epileptic seizures, this work presents a lightweight student model named RGF-Model. The model integrates a ring buffer storage mechanism [24], a GRU-based memory [21], and a FiLM-based dynamic gating strategy [25], together with a lightweight convolutional front end [42] and a Transformer encoder module [43], thereby enforcing causal consistency by using only past information. Within a knowledge distillation framework, RGF-Model distills knowledge from multiple teachers while maintaining low computational cost. The lightweight convolutional front end and Transformer modules in RGF-Model are informed by the feature extraction strengths demonstrated by the CNN [10] and Transformer [8] teachers. Furthermore, the Transformer models global dependencies within a 2-s window, whereas the GRU captures long-range historical dependencies across windows using a ring buffer for incremental updates, thereby supporting temporal information accumulation. This combination enables simultaneous capture of global patterns within short windows and long-term temporal evolution. The FiLM-based task modulation allows adaptive adjustment of feature representations for detection and prediction, thereby incorporating cross-task guidance from the teachers.

After data preprocessing, signal segments extracted by the sliding window were first fed into the lightweight convolutional front end for preliminary feature extraction. The lightweight convolutional front end comprised a Conv Stem layer and two MBConv layers. The Conv Stem applied a depthwise convolution with kernel size 3, followed by a 1 × 1 pointwise convolution to expand the single-channel input into 16 feature maps. A fixed lightweight configuration was used across experiments, with the Conv Stem producing 16 output channels, MBConv-1 using an expansion ratio of 4 and outputting 24 channels, and MBConv-2 using an expansion ratio of 4 and outputting 32 channels. The Transformer encoder had two layers with hidden size 64, feedforward dimension 128, four attention heads, and dropout 0.1. Subsequently, the signal was passed through the MBConv module for further processing. The channel expansion operation in the MBConv module is defined as in Equation (10).

(10) $$U_{p,q,c_{e}}=\sum_{c=1}^{C_{in}}X_{p,q,c}\cdot w_{c,c_{e}}^{\left(e\right)}+b_{c_{e}}^{\left(e\right)}.$$

In Equation (10), the spatial position of the input feature map X is indexed by p and q, whereas c and $c_{e}$ denote the input channel index and the expanded channel index, respectively. Weight $w_{c,c_{e}}^{\left(e\right)}$ and bias $b_{c_{e}}^{\left(e\right)}$ are the parameters of the channel-expansion convolution, and $U_{p,q,c_{e}}$ denotes the expanded intermediate feature map. This operation increases the channel dimensionality to capture richer signal features. Subsequently, a 3 × 1 depthwise convolution is applied to extract local spatial information from the feature map, as shown in Equation (11).

(11) $$V_{p,q,c_{e}}=\sum_{i=1}^{3}U_{P+i-1,q,c_{e}}\cdot w_{i,c_{e}}^{\left(dw\right)}.$$

In Equation (11), $w_{i,c_{e}}^{\left(dw\right)}$ denotes the weights of the depthwise convolution kernel, and $V_{p,q,c_{e}}$ denotes the features obtained after depthwise convolution. Channel compression is subsequently performed, as shown in Equation (12).

(12) $$Z_{p,q,c_{out}}=\sum_{c_{e}=1}^{C_{exp}}V_{p,q,c_{e}}\cdot w_{c_{e},c_{out}}^{\left(c\right)}+b_{c_{out}}^{\left(c\right)}.$$

In Equation (12), $w_{c_{e},c_{out}}^{\left(c\right)}$ and $b_{c_{out}}^{\left(c\right)}$ denote the weight and bias parameters of the channel-compression convolution, respectively. Finally, a residual connection X+Z was applied to produce the final output features. Subsequently, the Transformer encoder modeled the global relationships among these features. First, the stage 4 feature sequence was linearly projected into the query, key, and value matrices. This process is defined as in Equation (13).

(13) $$Q=XW_{Q},\;K=XW_{K},V=XW_{V}.$$

$W_{Q}$, $W_{K}$, and $W_{V}$ denote the query, key, and value projection matrices, respectively. Next, the similarity between the query and key is computed and scaled, as defined in Equation (14).

(14) $$S=\frac{QK^{T}}{\sqrt{d}}.$$

In Equation (14), d denotes the feature dimensionality of the attention mechanism, and the scaling factor $\sqrt{d}$ is introduced to prevent similarity values from becoming excessively large, thereby ensuring numerical stability during model training.

Next, softmax normalization is applied to each row of S to obtain the attention weight matrix A. Each element in the attention weight matrix A represents the relative importance among different positions in the feature sequence, with all weights summing to 1. Finally, the value matrix V is weighted and summed with the attention weights A to produce the output O. The output feature matrix O integrates information from all positions in the feature sequence and exhibits strong capability in capturing global temporal dependencies.

Along the temporal dimension, the model maintains a Ring Buffer $B_{t}$ of length L to store the features from the most recent L time steps. The update rule for the buffer sequence is defined in Equation (15).

(15) $$B_{t}=\left[F_{t-L+1},F_{t-L+2},\cdots ,F_{t}\right].$$

Here $F_{t}$ denotes the feature representation produced by the Transformer at time step t. The ring buffer captures recent feature dynamics by continually updating its stored history. In this study, the Transformer operates only on token sequences within a single 2-s window, and its output is passed to the GRU to accumulate information across windows along the temporal axis. On write, the pointer advances by one slot per step and wraps around, storing the current hidden state at the current position and overwriting the oldest entry. On read, a fixed-weight linear aggregation over the most recent L hidden states yields a temporal summary. The per-step read and write cost remains constant and does not increase with L. Moreover, the ring buffer causally aggregates the most recent L hidden states into the current decision, suppressing short-term fluctuations in per-window outputs and extending the duration of positive decisions. This temporal smoothing consolidates sporadic boundary positives into sustained above-threshold alarms without increasing false alarms, thereby improving Sen under a specified FPR/h constraint.

To maintain consistency with deployment-side temporal smoothing, the ring buffer length L was aligned with the post-processing voting window n (default n=10 covering approximately 10 s of near-term memory). During inference, streaming updates were used in which the current hidden state was written to the buffer pointer’s position, and the pointer incremented modulo L, overwriting the oldest slot. For reading, a lightweight aggregation over the most recent L states yielded the temporal summary $s_{t}$. At model start-up, L cold-start fills were performed to stabilize memory. This design ensures strict causality and a fixed memory footprint, facilitating on-device deployment.

Subsequently, the GRU memory network integrates the previous hidden state $h_{t-1}$ with the current input $x_{t}$ through a gating mechanism, thereby progressively updating the hidden state $h_{t}$. The update gate is defined in Equation (16), the reset gate in Equation (17), the candidate hidden state in Equation (18), and the final hidden state in Equation (19).

(16) $$z_{t}=\sigma \left(W_{z}x_{t}+U_{z}h_{t-1}+b_{z}\right),$$

(17) $$r_{t}=\sigma (W_{r}x_{t}+U_{r}h_{t-1}+b_{r}),$$

(18) $${\tilde{h}}_{t}=\tanh(W_{h}x_{t}+U_{h}(r_{t}⊙h_{t-1})+b_{h}),$$

(19) $$h_{t}=z_{t}⊙h_{t-1}+\left(1-z_{t}\right)⊙{\tilde{h}}_{t}.$$

In the Equation, the GRU gating mechanism effectively controls the retention and updating of historical information, enabling hidden states to accumulate long-term memory adaptively.

After obtaining the hidden state $h_{t}$ from the GRU module, the model incorporates the FiLM mechanism to achieve task-specific feature modulation for epilepsy seizure prediction and detection. Specifically, modulation coefficients are defined separately for the prediction and detection tasks, and linear transformations are applied to the hidden states on a channel-by-channel basis through the FiLM module. The modulation calculation for the detection-task features is defined in Equation (20).

(20) $$D_{t,c}=\gamma_{c}^{\left(det\right)}\cdot h_{t,c}+\beta_{c}^{\left(det\right)}.$$

In Equation (20), $D_{t,c}$ denotes the value of the detection-task feature after FiLM modulation at time step t in channel c. $\gamma_{c}^{\left(det\right)}$ and $\beta_{c}^{\left(det\right)}$ denote the linear scaling and offset parameters of channel c in the detection branch, respectively. These two parameters are generated by a lightweight fully connected network based on the detection probability output from the previous time step and are used to dynamically adjust feature scaling and bias along the channel dimension. $h_{t,c}$ denotes the value of the GRU hidden state $h_{t}$ in channel c. This modulation method adaptively emphasizes or suppresses specific features in certain channels based on feedback from the detection branch, thereby better accommodating the requirements of the detection task.

Similarly, the modulation calculation for predicting task features is shown in Equation (21).

(21) $$P_{t,c}=\gamma_{c}^{\left(pred\right)}\cdot h_{t,c}+\beta_{c}^{\left(pred\right)},$$

where $P_{t,c}$ denotes the value of the prediction-task feature after FiLM modulation at time step t in channel c. $\gamma_{c}^{\left(pred\right)}$ and $\beta_{c}^{\left(pred\right)}$ denote the linear scaling and offset parameters of channel c in the prediction branch, respectively, which are generated by a lightweight fully connected network designed for the prediction task. In this manner, the prediction branch dynamically adjusts the feature representation of each hidden-state channel according to the characteristics of the current task, thereby enabling the model to more effectively capture information related to future states.

To enhance the Sen of the prediction branch to weak signals in the early stages of an event and to avoid the use of future information under causal constraints, the output probability of the detection branch at time t is employed as a SFPM, and exponential smoothing is applied to obtain a scalar prior that depends solely on current and historical information, as defined in Equation (22).

(22) $$s_{t}=\lambda s_{t-1}+(1-\lambda )y_{t}^{det}.$$

Among them, $s_{0}=y_{0}^{det}$ is the initialized value, λ∈[0.85,0.95] and $y_{t}^{det}$ denote the occurrence probabilities of the detection branch at time step t. During training, stop-gradient processing is applied to $s_{t}$, and amplitude regularization is imposed on the subsequently generated modulation coefficients to suppress positive-feedback amplification caused by false alarms. During inference, the same data flow and smoothing strategy are employed to ensure consistency with online deployment.

Given condition $s_{t}$, we generate channel-level FiLM parameters $\gamma_{t}^{pred}$ and $\beta_{t}^{pred}\in R^{C}$ using a two-layer perceptron ∅prior (⋅), which are then employed to linearly modulate the GRU hidden state $h_{t}\in R^{C}$. The task-specific representation of the prediction branch is derived as presented in Equation (23).

(23) $${\tilde{h}}_{t}^{pred}=(1+\gamma_{t}^{pred})⊙h_{t}+\beta_{t}^{pred}.$$

Among them $\left[\gamma_{t}^{pred},\beta_{t}^{pred}]=\emptyset \mathrm{prior}(s_{t}\right)$, ⊙ denote channel-wise multiplication. The resulting ${\tilde{h}}_{t}^{pred}$ is used as input to generate $y_{t}^{pred}$, serving as the prediction header. This design enables “detection-to-prediction” SFPM within a unified network, while rigorously maintaining causal order. The SFPM was derived from the detection branch’s temporal probabilities using an exponential moving average and truncated normalization, then was scaled by a coefficient λ in the range 0.85 to 0.95 and injected as a read-only conditioning variable into the prediction branch. To prevent runaway positive feedback, gradients were not back propagated from the prior pathway to the detection branch, and temporal smoothing and an upper bound constraint were applied to the prior sequence.

Finally, the model transmits the features generated by the FiLM module to two independent output branches—detection and prediction—thereby enabling real-time seizure detection and future event prediction. Among them, the detection branch utilizes the modulated detection feature $D_{t}$ to compute the detection result at the current time, as presented in Equation (24).

(24) $${\hat{y}}_{t}^{\left(det\right)}=f_{det}(D_{t}).$$

In Equation (24), ${\hat{y}}_{t}^{\left(det\right)}$ denotes the detection result produced by the model at time t, indicating whether a seizure is identified within the current window. Function $f_{det}(\cdot )$ serves as the output function of the detection branch, typically implemented as a mapping network comprising one or more fully connected layers, which maps FiLM features to the final result space of the detection task. The detection results enable real-time alerts, facilitating timely seizure detection.

The prediction branch estimates the probability of an epileptic event within a specified future time window by utilizing the modulated prediction feature $P_{t}$. The specific process is illustrated in Equation (25).

(25) $${\hat{y}}_{t}^{\left(pred\right)}=f_{pred}\left(P_{t}\right),$$

where ${\hat{y}}_{t}^{\left(pred\right)}$ denotes the predicted probability of a future epileptic seizure produced by the model at time t, reflecting the likelihood of an event within a forthcoming prediction window. Function $f_{pred}(\cdot )$ serves as the output function of the prediction branch, typically implemented as a mapping network comprising one or more fully connected layers, which maps FiLM features to the final probability space required for the prediction task. The prediction branch outputs an early warning signal, enabling timely intervention and preventive measures.

**Figure 4:**
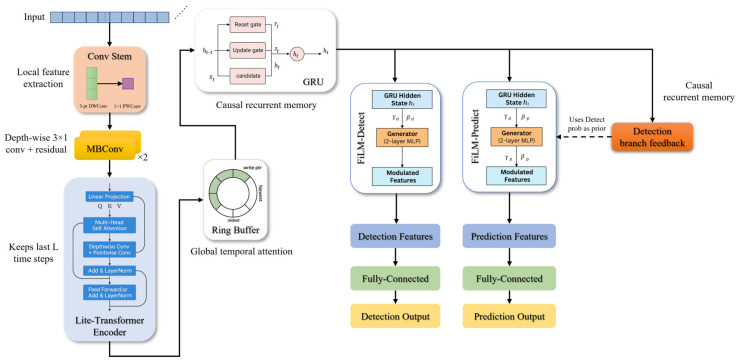
RGF-Model Structure Diagram.

In summary, through task-specific FiLM modulation of GRU hidden-state features, the RGF-Model integrates prediction and detection requirements, enabling a shared backbone while preserving task-specific representations. This enables real-time joint processing of prediction and detection and improves the practical utility and clinical effectiveness of epilepsy monitoring. The architecture of the RGF-Model is shown in Figure 4.

Regarding system initialization, often termed a cold start, the Ring-Buffer mechanism inherently necessitates an accumulation period equivalent to the buffer size L to establish a valid probability distribution. Consequently, the detector naturally refrains from generating alarms during this initial phase until sufficient temporal context is available, which effectively functions as a warm-up period to ensure stability. As for signal disruptions including sensor dropouts, the preprocessing pipeline described in Section 2.2 serves as the primary defense against artifacts through band-pass filtering. In a practical clinical deployment, this strategy would be further augmented by standard impedance monitoring to suppress outputs during instances of complete electrode detachment.

### 2.5. Multi-Teacher Knowledge Distillation Strategy

To build a lightweight model for seizure prediction and detection, a multi-teacher knowledge distillation framework was employed. Two sets of high-performing teacher models were first trained, one for seizure prediction and the other for real-time detection. During distillation, one teacher from each set was paired with the student to form a multi-teacher configuration. Pairs of teachers were randomly sampled within mini-batches to iteratively cover all teachers, and parallel averaging across teachers was avoided to reduce gradient instability. The student’s detection branch was supervised with discriminative guidance and soft labels from the selected detection teacher, whereas the prediction branch was guided to capture preictal temporal dynamics by the selected prediction teacher. This design enabled the effective transfer of knowledge distilled from the teachers. The two teachers were trained independently on their respective tasks. For detection, cross-entropy with class-frequency weighting was used. For prediction, cross-entropy was used with labels defined by a 30-min preictal window. Early stopping and hyperparameters were selected on each task’s validation set. This configuration yielded complementary inductive biases and attention scales between the two teachers.

During distillation for the seizure prediction task, the student’s prediction branch captured the temporal dynamics of preictal features from the ensemble of prediction teachers. During distillation for the seizure detection task, the student’s detection branch acquired soft labels, namely the prediction probability distribution, from the ensemble of detection teachers for supervision. Additionally, a temporal consistency regularization was incorporated in prediction distillation to guide the student in learning the temporal evolution of the teachers’ prediction probabilities. Specifically, when the teachers’ prediction probabilities increase as seizure onset approaches, the student was encouraged to exhibit a corresponding upward trend. This constraint enabled the student not only to match the teachers’ instantaneous outputs but also to replicate the temporal evolution of their predictions, thereby capturing gradually emerging preictal signals. The process is defined in Equation (26).

(26) $$L_{\mathrm{TimeReg}}=\frac{1}{T-1}\sum_{t=1}^{T-1}{\left[\left({\hat{p}}_{t+1}-{\hat{p}}_{t})-(p_{t+1}-p_{t}\right)\right]}^{2}.$$

In Equation (26), $P_{t}$ and $P_{t+1}$ denote the prediction probabilities produced by the tteacher at time steps t and t+1, respectively, whereas ${\hat{p}}_{t}$ and ${\hat{p}}_{t+1}$ represent the corresponding probabilities generated by the student. Feature alignment for the detection teacher was applied only to the student detection branch (after FiLM-det). In contrast, alignment for the prediction teacher was applied only to the student prediction branch (after FiLM-pred). The shared backbone (Conv, Transformer, GRU) did not undergo feature alignment and instead received only supervision and soft labels, which mitigated gradient conflicts between the two tasks.

**Figure 5:**
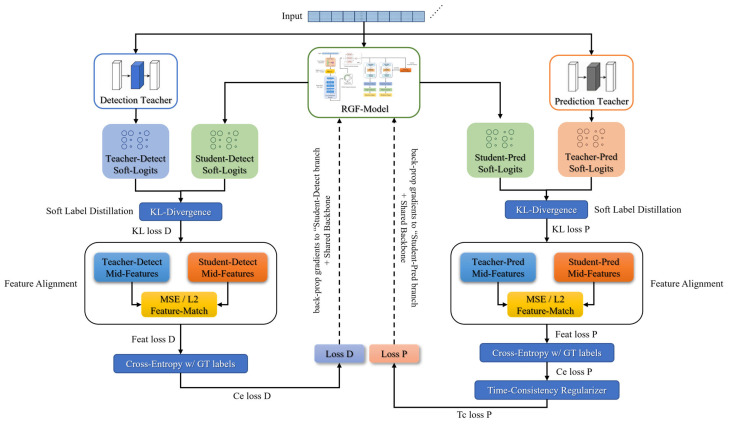
Knowledge Distillation Strategy Structure Diagram.

With this constraint, the student not only matched the teachers’ outputs at individual time points but also captured the patterns underlying the gradual change in prediction probabilities preceding a seizure. Each parameter update minimized both the supervised loss and the distillation loss. The supervised loss comprised the cross-entropy of the prediction branch and the detection branch, and the distillation loss comprised matching temperature-softened output distributions and aligning intermediate features on the corresponding branches. The prediction branch additionally included a temporal consistency constraint, and the SFPM from detection was injected into prediction in a read-only manner. All components were combined as a weighted sum with fixed coefficients selected on the validation set and held constant across experiments. For samples labeled for a single task, the terms related to the other task were masked and excluded from the backpropagation process. To avoid conflicts among multiple teachers, distillation operated only on the corresponding branch, and the teacher networks did not receive gradients from the student. The specific structure is illustrated in Figure 5.

Temperature-softened logit distillation was combined with intermediate feature alignment. The temperature was set to τ=2 by default, and small-scale validation over {1, 2, 4} showed that τ=2 was stable on both datasets. The total loss was a weighted sum of the task supervision loss and the two distillation terms. For samples labeled for a single task, distillation terms for the unlabeled task were masked and excluded from backpropagation. Complementarity among teachers arose from differences in architecture and focus scales. Prediction teachers emphasized slowly evolving preictal cues, whereas detection teachers highlighted burst rhythms and spike-and-wave patterns. To avoid conflicts, the teacher networks did not receive gradients from the student, and the two distillation streams operated only on their corresponding task-modulation branches. Teacher outputs with low confidence were temperature-softened and down-weighted to reduce the impact of inconsistent guidance on the student.

Furthermore, the FiLM-based self-feedback mechanism embedded within the student enhanced its capacity to capture preictal state changes, thereby complementing temporal consistency regularization and improving prediction performance. Finally, the distillation loss was integrated with a conventional supervised loss based on cross-entropy with the true labels, defining the overall training objective within the knowledge distillation framework. No features explicitly dependent on future time steps were distilled, thereby ensuring strict causality of the student network.

### 2.6. Training and Loss Function

This study adopted a unified training and validation strategy for the dual tasks of seizure prediction and detection, with the goal of comprehensively evaluating the model’s generalization. Each subject was modeled separately, and performance was evaluated using leave-one-out cross-validation (LOOCV) [44]. Specifically, each patient’s EEG data were partitioned into multiple paired seizure and non-seizure segments based on seizure events. In each round, one pair was held out for testing, and the remaining pairs were used for training and the procedure was repeated until every pair had served once as test data. To reduce variability due to differences in seizure frequency and recording duration among participants and to maintain comparability with prior studies, this study computed the unweighted arithmetic mean of all metrics across participants after individual-level LOOCV evaluation, and reported this mean as the final result. This approach provided a robust estimate of the model’s overall generalization capability, reduced the undue influence of individuals with very few seizures or unusually long recordings, and better reflected the model’s average expected performance in real-world, clinically diverse populations. Because each participant underwent the same training and testing process and averaging did not alter the relative ranking of individual metrics, reporting averages across participants neither obscured individual-level performance trends nor compromised the validity of the ablation study conclusions.

Regarding the training objective, the model was required to perform both seizure prediction and detection. Therefore, the loss function incorporated supervision and knowledge transfer for both tasks. For the detection branch, cross-entropy with ground-truth labels was used, and soft labels from the detection teacher were incorporated through a distillation term to enhance the student’s discriminative capability. The prediction branch likewise used cross-entropy with ground-truth labels together with a distillation loss derived from the prediction teacher. Additionally, a temporal consistency regularization was included to encourage smoothness and trend consistency in prediction outputs over time. Together with the knowledge distillation framework described in Section 2.5, the overall training objective is formulated as in Equation (27).

(27) $$L=\alpha (L_{det}^{CE}+L_{pred}^{CE})+\beta (L_{det}^{KD}+L_{pred}^{KD})+\gamma L_{pred}^{TimeReg}.$$

Among them, $L_{det}^{CE}$ and $L_{pred}^{CE}$ denote the cross-entropy supervision losses for the detection and prediction tasks, respectively, whereas $L_{det}^{KD}$ and $L_{pred}^{KD}$ represent the knowledge distillation losses for the corresponding task branches, applied to the post-FiLM representations within each branch. $L_{pred}^{TimeReg}$ corresponds to the temporal consistency loss for the prediction branch. Hyperparameters α, β, and γ are introduced to weight the supervision loss, distillation loss, and temporal consistency constraint in the total loss, respectively, thereby achieving an optimal trade-off between knowledge transfer and task performance during training.

To mitigate overfitting due to limited data, the last 25 percent of the training set was held out as a validation set, and early stopping was employed. If the validation loss did not decrease for 10 consecutive epochs, training was halted, and the parameters at the epoch with the lowest validation loss were retained.

### 2.7. Postprocessing

To improve stability and clinical applicability in real-world settings, post-processing strategies tailored to seizure prediction and detection were devised. For seizure prediction, k-of-n multiwindow decision scheme with a refractory period was adopted to reduce the false positive rate, following prior work [45]. Specifically, an alarm was issued only when at least k=8 of the most recent n=10 prediction windows were positive, for example, five consecutive minutes comprising ten 30-s windows. Additionally, a 30-min refractory period was imposed after each alarm to prevent multiple alarms within a short interval. During this period, no additional alarms were generated even if the outputs again satisfied the alarm condition. This strategy suppresses spurious false alarms and enhances the reliability and clinical usability of the warning system.

For seizure detection, the model was required to classify seizure states in real time within each EEG window. However, due to noise and transient anomalies, classification models can produce false positive alarms in very short windows. Therefore, a window-level smoothing alarm mechanism was introduced [46]. Specifically, the detection probabilities from consecutive windows were smoothed with a moving average. An alarm was triggered only when the averaged detection probability over a consecutive 5-s interval exceeded a predefined threshold, thereby filtering false alarms caused by transient fluctuations. This smoothing strategy preserves real-time performance and Sen and reduces the false positive rate, thereby enhancing the system’s practicality and usability. Together, the prediction and detection tasks and the post-processing mechanisms provide a solid foundation for reliable automated analysis of epileptic seizures.

### 2.8. Experimental Environment

Training used the Adam optimizer with an initial learning rate of 0.01 and a batch size of 32. All experiments were conducted in Python 3.9 with PyTorch 1.10.0 (CUDA 11.6) on an NVIDIA GeForce RTX 3090 Ti GPU (NVIDIA Corporation, Santa Clara, CA, USA) and an Intel Core i9-10900X CPU (Intel Corporation, Santa Clara, CA, USA). A 2 s sliding window with a 1 s stride was used for modeling across all experiments. Post processing used a k-of-n voting scheme with window size n=10, and the ring buffer length was set to L=10. The SFPM from detection to prediction was obtained by applying an exponential moving average with smoothing coefficient α=0.6 to detection probabilities, then scaling by a magnitude coefficient in the range 0.85 to 0.95 with default λ=0.9, and injecting the result as a read only conditioning variable into the prediction branch. The coefficient was tuned on the validation set. During online inference, the model maintained state across time steps via a ring buffer. At each new time step, the current hidden state was written to the current buffer position, and the write pointer advanced by one slot. When the pointer reached the end, it wrapped to the beginning and overwrote the oldest record. This mechanism involved a fixed number of read and write operations per step, incurred constant computational and memory cost, and had update time independent of the buffer length, which ensured stable inference efficiency and system latency.

## 3. Results

This section details the experimental protocol and results. The evaluation included cross dataset transfer experiments and mixed dataset tenfold cross validation, ablations on FiLM, Transformer temporal encoding, the detection to prediction prior, temporal consistency regularization, and the use of multi teacher, single teacher, and no distillation settings, robustness tests with repeated random seeds, comparisons with the teacher models and recent methods, on device efficiency under a unified CPU benchmark, and Sen analyses of the k-of-n rule and the refractory period to substantiate the performance of RGF-Model.

### 3.1. Experimental Settings

**Figure 6:**
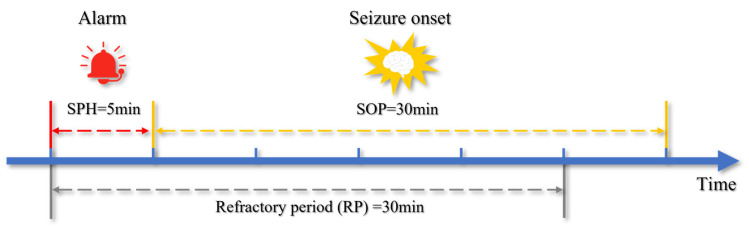
Description of the seizure occurrence period (SOP) and seizure prediction horizon (SPH).

In seizure prediction, the seizure prediction horizon (SPH) and the seizure occurrence period (SOP) are first defined. The SOP denotes the period during which a seizure is expected to occur, whereas the SPH is the interval between issuance of the warning signal and the onset of the SOP, as illustrated in Figure 6. A prediction is valid when the model issues a warning within the SPH before the seizure, and the seizure occurs within the subsequent SOP. Consistent with mainstream literature, the preictal label in the training data was aligned with an SOP of 30 min, whereas an SPH of 5 min was used only to evaluate trigger timing. This design ensures sufficient time for transfer or intervention and avoids prolonged alarms that may cause unnecessary anxiety [32]. After an alarm was issued, a 5-min SPH elapsed first and was followed by a 30-min SOP. If the onset occurred within that SOP, the alarm was counted as valid; otherwise, it was invalid. For consistency, a 30-min preictal window was used for labeling in both training and evaluation, whereas the 5-min SPH was applied only to assess alarm timing and suppress spurious early alarms, and it was not used to generate training labels or to compute any loss terms.

Model performance was evaluated using four metrics: the AUC, Sen, the FPR/h, and statistical significance against a random predictor. Among these metrics, AUC measures separability between seizure and non-seizure episodes, Sen measures the proportion of seizures correctly predicted relative to all episodes, and FPR/h quantifies the average number of false alarms per hour computed over the complete unedited test duration. To quantify performance variability across subjects, all aggregated experimental results are reported as mean ± standard deviation (SD). Additionally, statistical significance was assessed. Specifically, the probability that a random predictor would trigger an alarm by chance during the SOP was estimated from the model FPR, and the probability that this random predictor would achieve at least as many successful predictions as the proposed model across all seizures was then computed. For each subject, if this probability was less than 0.05, the predictive capability of the proposed model was considered significantly superior to that of a random predictor. Crucially, to avoid the statistical inaccuracy of averaging p-values, we report the specific p-values for individual subject analysis (e.g., in cross-dataset experiments) and the Significant Ratio (abbreviated as Sig. (%) in tables) for aggregated group-level results. The Significant Ratio is defined as the percentage of subjects for whom the predictive capability was significantly superior to that of a random predictor (p < 0.05) [32].

For seizure detection, evaluation metrics include Acc, Sen, Specificity (Spe), and AUC [47], which together assess recognition performance and the ability to limit false positives. These settings provided a comprehensive, objective, and reproducible evaluation of the lightweight RGF-Model in dual task scenarios.

To evaluate the model’s lightweight characteristics, two metrics were used: Parameters (Params) and Model Size (MB). Params denote the total number of trainable weights, reflect structural complexity, and are reported in millions (M). Model Size denotes the required storage, computed from the weight quantization bit width, and reported in MB. Smaller values indicate a lighter model and improve suitability for deployment on portable devices.

### 3.2. Experimental Results

By pairing teachers of different architectures and adopting a multi-teacher knowledge distillation strategy, the performance of the lightweight RGF-Model was systematically evaluated on the CHB-MIT and Siena EEG datasets for both seizure prediction and detection. Results showed that the student achieved feature transfer and soft label supervision from both prediction and detection teachers and integrated ground truth labels for end-to-end optimization, thereby balancing knowledge transfer with task adaptability. All models were constructed with unified input channel counts and window lengths, while each dataset retained its original sampling rate. Only the input dimensions were adjusted to accommodate different sampling rates. Table 3, Table 4, Table 5 and Table 6 present performance metrics for student models distilled with different teacher combinations, together with parameter counts and storage footprint, thereby demonstrating the lightweight and practical nature of the method.

Table 3 presents the performance of student models on CHB-MIT after distillation with different teacher combinations for the prediction task. The results show that the student models achieve strong predictive performance regardless of the teacher combination used. The average AUC reaches 98.66%, Sen consistently exceeds 98%, and FPR/h remains between 0.01 and 0.05. Among the configurations, the student distilled from the multidimensional Transformer as the prediction teacher and CNN-LSTM with attention as the detection teacher achieves the best performance, with AUC 99.54%, Sen 98.71%, and FPR/h 0.01. This suggests that the student effectively captures preictal patterns while maintaining a very low false positive rate. Notably, the parameter counts of all student models range from 0.06 to 0.09 M, with model sizes of only 0.24 to 0.36 MB. Maintaining such high performance at this compact scale demonstrates that multi-teacher knowledge distillation enhances model efficiency and compactness and supports clinical applications and deployment on portable devices. Additionally, the performance distribution across teacher combinations is balanced, with no evident outliers, which further confirms the robustness of the training process and the effectiveness of complementary multi-teacher distillation.

Table 4 presents the performance of student models on the Siena dataset after distillation with different teacher combinations for the prediction task. Overall, the Siena results maintain the high performance observed on CHB-MIT, with an average AUC of 97.56% for the student models. The optimal teacher combination achieves AUC 98.97%. All combinations achieve AUC above 96%. Sen ranges from 96% to 99%, and FPR/h remains low at 0.02 to 0.06, which further demonstrates strong generalization. Moreover, parameter counts and model sizes are consistent with those on CHB-MIT, reflecting cross-dataset generalizability and lightweight advantages. Overall, the student achieves a favorable balance between high performance and low resource consumption in the prediction task, demonstrating strong transferability and practical applicability.

Table 5 presents the performance of student models on CHB-MIT after distillation with different teacher combinations for the seizure detection task. The results show that Acc exceeds 97% across all teacher combinations, Sen and Spe are approximately 98%, and AUC is generally above 98% with some configurations approaching 99%. These results indicate high performance and robustness. Additionally, the student models have parameter counts of 0.06 to 0.09 M and model sizes of 0.24 to 0.36 MB. This shows that multi-teacher knowledge distillation reduces model complexity while preserving task performance, enabling flexible application and feasible deployment by integrating knowledge from multiple teachers.

Table 6 presents the overall performance of student models for the seizure detection task on the Siena dataset after applying a multi-teacher knowledge distillation strategy. The results show that Acc is not lower than 98.62%, Sen remains between 98% and 99%, Spe is not lower than 98.5%, and AUC stays above 98.6%, with some combinations approaching 99%. Performance differences across teacher combinations are small, indicating robustness and consistency. Overall, the multi-teacher distillation framework achieves high Acc, a lightweight design, and deployment friendliness on Siena, showing promising applicability in resource-constrained settings.

**Figure 7:**
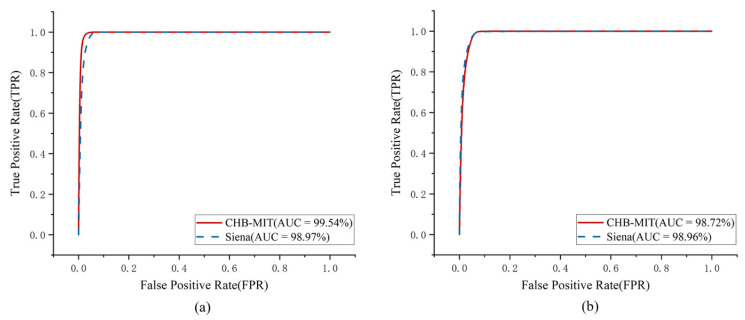
(**a**) ROC curves of the RGF-Model for seizure prediction on the CHB-MIT and Siena datasets; (**b**) ROC curve of the RGF-Model for seizure detection on the CHB-MIT dataset and Siena datasets.

As an illustrative case, the student distilled from the multidimensional Transformer and the CNN-LSTM with attention exhibited strong performance. On the CHB-MIT and Siena datasets, this model produced near-perfect ROC curves for seizure prediction and detection, as shown in Figure 7. All curves lie close to the upper left corner of the coordinate plane, indicating very high Sen and Spe. Across subjects, the standard deviation of each metric ranged from 1% to 3%. For example, on CHB-MIT, AUC varied within ±1.5% across subjects. These results indicated stable performance across patients without catastrophic failures in individual cases.

**Figure 8:**
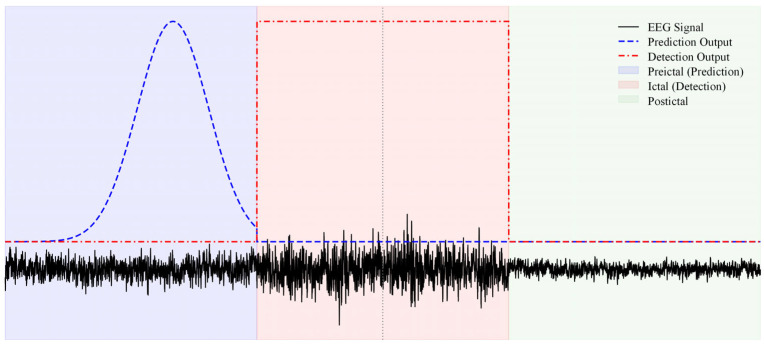
Model Prediction and Detection Outputs on EEG Signals Across Different Seizure Stages.

Experimental results indicate that the model effectively performs real-time seizure prediction and detection from EEG signals across varying time intervals. Figure 8 shows that the prediction output rises markedly during the preictal phase, enabling an early response to seizure risk, whereas the detection output quickly identifies the ictal interval once the seizure begins. These results further support the effectiveness of the method for time series signal processing and multistage epilepsy recognition.

Saliency and temporal output analyses indicate that the model’s attention patterns are broadly aligned with established EEG phenomena. On the detection side, the model shows stronger responses to abrupt ictal features such as rhythmic discharges, spikes, and spike and wave complexes, and is accompanied by rapid increases in short-term energy and spectral power. On the prediction side, the model is more sensitive to slow and sustained preictal changes, including gradual increases in low to mid frequency power, attenuation of background rhythms, especially the alpha rhythm, strengthened cross-channel synchrony, and a tendency for intermittent spikes to cluster. Overall, the model exploits two complementary cues, gradual time frequency changes during the preictal period and abrupt rhythmic transitions during the ictal period, which accord with electrophysiological literature on preictal and onset patterns and align with the design goals of causal memory and task modulation. These observations provide an intuitive basis for understanding how the model extracts discriminative preictal and ictal features across time and frequency.

Overall, the multi-teacher knowledge distillation strategy combined with the lightweight RGF-Model demonstrated strong performance and adaptability in seizure prediction and detection. Results on the CHB-MIT and Siena datasets showed that, while maintaining low model complexity, the student consistently achieved high Sen, low FPR/h, high Acc, and high AUC. These results indicate strong adaptability of the student across tasks and the effectiveness of the multi-teacher distillation strategy, providing a foundation for practical deployment and engineering implementation of automatic seizure warning systems.

### 3.3. Ablation Experiment

A series of ablation experiments was designed to evaluate the independent contribution of each component to overall performance. Using the lightweight student network as the base, experiments on CHB-MIT and Siena examined the impact of FiLM task modulation, Transformer temporal encoding, a SFPM injected from detection to prediction, and temporal consistency regularization on prediction and detection performance. It should be noted that the ring buffer and the GRU jointly formed the basic framework for causal online inference and cross-window memory. Removing either would break real-time causality and hinder a fair comparison with the original lightweight baseline. Accordingly, no structural ablation was performed that removed either component. Additionally, to assess the effectiveness of the distillation strategy itself, three training configurations were compared under an unchanged student architecture, namely multi-teacher distillation, single teacher distillation, and no distillation. In the single teacher setting, soft supervision was applied only to the covered task; the other task was excluded from training, its metrics were not reported, and the network structure and parameter count matched the multi teacher setting.

In the ablation experiments, the multidimensional Transformer served as the prediction teacher and the CNN-LSTM with attention served as the detection teacher, selected for their strong performance and complementary architectures. The multidimensional Transformer excelled at global modeling and long-range dependency analysis, effectively capturing subtle preictal dynamics, whereas the CNN-LSTM with attention focused on local feature extraction and temporal dynamics. Their structural complementarity provided rich knowledge sources, and their clear intermediate layer representations facilitated efficient alignment-based distillation, improving the effectiveness of knowledge transfer.

Table 7 and Table 8 present ablation results on CHB-MIT and Siena. On CHB-MIT, Full KD achieved the best performance. Removing FiLM reduced prediction AUC from 99.54% to 96.42% (a decrease of 3.12 percentage points), lowered detection Acc from 98.78% to 96.52%, and reduced detection AUC to 96.88%, indicating that FiLM plays a key role in task-specific representation modulation. After removing the Transformer, prediction AUC was 97.27% (2.27 percentage points lower than Full KD) and detection AUC was 96.79%, suggesting that long-range dependency modeling is secondary to FiLM yet still provides stable gains. Removing the detection to prediction SFPM yielded prediction AUC 97.12%, detection Acc 98.55%, and detection AUC 98.05%, indicating contributions to false alarm suppression and cross window consistency.

When temporal consistency regularization was removed, prediction AUC and Sen decreased to 98.26% and 98.11%, and detection Acc and AUC were 97.69% and 98.08%, slightly below Full KD, indicating that this regularizer helps smooth prediction sequences and reduce output fluctuations. For the distillation strategy, single teacher distillation using only the prediction teacher produced a prediction AUC of 98.92%, 0.62 percentage points below multi teacher, and increased FPR/h from 0.01 to 0.02. Using only the detection teacher yielded detection Acc 98.45% and detection AUC 98.36%, 0.33 and 0.44 percentage points lower than Full KD. With distillation removed, both tasks degraded, with prediction AUC 95.78%, Sen 95.21%, FPR/h increased to 0.07, and detection Acc and AUC dropped to 96.12% and 96.10%, with differences statistically significant. Overall, a clear performance ordering emerged in which multi-teacher distillation outperformed single teacher and single teacher outperformed no distillation, further validating the value of multi-source knowledge fusion for improving performance and stability.

On the Siena dataset, results mirrored those on CHB-MIT. Full KD achieved the best overall performance. Removing FiLM significantly reduced both prediction and detection metrics, highlighting its role in task-specific modeling. Excluding the Transformer, which caused moderate drops, suggests its secondary yet beneficial role in capturing long-range dependencies. Omitting the detection-to-prediction SFPM reduced prediction AUC and weakened cross-window consistency. Without temporal regularization, performance slightly declined, showing its value in stabilizing outputs. Using only the prediction or detection teacher led to modest reductions compared to multi-teacher KD. Without distillation, performance dropped sharply, with increased false alarms and decreased accuracy, underscoring the importance of soft label guidance and cross-task learning. These results reaffirmed the contributions of each module and the effectiveness of the distillation strategy, supporting the method’s robustness and generalizability.

The ablation results underscored the distinct functional roles of each module in the lightweight student model. FiLM enabled task-aware, channel-wise feature modulation, allowing adaptive separation and optimization for prediction and detection, thus enhancing overall performance-consistent with its established role in cross-task generalization [25]. The Transformer encoder captured long-range dependencies and complemented the GRU’s limited short-term memory, aligning with prior findings on its utility in seizure prediction [48]. The detection-to-prediction SFPM provided real-time feedback, increasing sensitivity to weak preictal signals and supporting shared representations for joint task improvement [49]. Temporal consistency regularization stabilized output probabilities over time, improving smoothness, in line with structural regularization benefits in sequence modeling [50].

The distillation strategy followed a clear performance trend. Multi-teacher distillation, by integrating complementary supervision from prediction and detection teachers, achieved better calibration, lower variance, and the best overall performance. Single-teacher setups mainly benefited the corresponding task, while the other degraded. Removing distillation led to significant performance drops and higher FPR/h, confirming the value of shared representations in multi-task learning [49].

Ablation and temporal output analysis showed FiLM yielded more stable, confident predictions in preictal windows. Combined with the ring-buffer GRU, outputs became smoother and alarms more continuous, reflecting the gradual preictal evolution and abrupt ictal onset. Together, these components improved effective alarm rates without increasing FPR/h, explaining the observed gains in sensitivity. In sum, the experiments quantitatively validated each module’s contribution and confirmed the efficacy of the modular design and multi-teacher distillation strategy, supporting its application in lightweight, real-time seizure prediction and detection.

### 3.4. Cross Subject and Cross Dataset Generalization Experiments

To assess the generalization of the RGF Model to unseen subjects and distribution shifts, five complementary experiments were designed and executed without altering the established preprocessing, model architecture, or post-processing. All experiments used a grouped ten-fold cross-validation or cross-dataset one-way transfer and strictly prevented information leakage. Folds were defined at the subject level, and data from a given subject never appeared in both training and validation or test. Thresholds and post-processing parameters were fixed on the training portion, whereas validation and test were used only for evaluation. In each fold, metrics were computed at the subject level, averaged across subjects, and aggregated across the ten folds as the mean and the standard deviation.

The first and second experiments performed a fold cross-validation on CHB-MIT and Siena, respectively. In each fold, data from subjects in nine folds were used to train the teacher and student models, and the remaining fold of subjects was used for prediction and detection evaluation. For prediction, AUC, Sen, and FPR/h were reported, whereas for detection, Acc, Sen, Spe, and AUC were reported. For each fold, metrics were first macro-averaged at the subject level and then aggregated across the ten folds as the mean and the standard deviation to quantify generalization to unseen subjects within the same dataset. The third and fourth experiments evaluated cross-dataset transfer. Training was performed on CHB-MIT with direct testing on Siena, and training was performed on Siena with direct testing on CHB-MIT.

During training, teacher and student fitting and threshold selection were completed on the source dataset. During testing, direct evaluation was conducted on the target dataset with no retraining or recalibration. To reduce bias from acquisition differences, cross-dataset evaluation used only the channels common to both datasets and a unified referencing scheme, with all other processing identical to the main experiments. On the target dataset, metrics were computed at the subject level and macro-averaged to characterize out-of-distribution generalization. The fifth experiment merged CHB-MIT and Siena and performed a grouped ten-fold cross-validation. Fold generation jointly considered subject identity and dataset origin so that, while subjects were mutually exclusive across folds, the sample proportions from the two datasets were kept as similar as possible in each fold. Training and evaluation followed the same procedure as above, and metrics were summarized across the ten folds as the mean and the standard deviation to reflect overall generalization to unseen subjects under mixed domain conditions.

As shown in Table 9, under strict cross-subject and cross-dataset evaluation, the RGF Model maintained stable and practically useful performance and exhibited strong generalization across settings. In single dataset cross-subject validation on CHB-MIT, AUC 93.81% and detection Acc 94.86% were obtained. In Siena, AUC 92.91% and Acc 94.23% were achieved, with FPR/h 0.10 and 0.11, respectively. Standard deviations across folds were small, indicating limited influence of subject splits and stable preictal and ictal recognition without sharing subject information. In the more challenging cross-dataset transfer, training on CHB-MIT and testing on Siena yielded AUC 87.04% and Acc 91.07%. Conversely, training on Siena and testing on CHB-MIT yielded AUC 84.57% and Acc 89.83%.

Although these values were lower than in the cross-subject setting, the magnitude remained reasonable, and *p* value < 0.05 indicated statistical significance, reflecting the impact of cross-center and cross-population distribution shift while showing that the model remained acceptable without target domain fine-tuning. Notably, after merging the two datasets and conducting mixed domain cross-subject validation, AUC increased to 95.09%, Acc increased to 95.71%, and FPR/h decreased to 0.09, with standard deviations remaining low, which supports that richer inter-individual and intra-domain diversity during training improves robustness to unseen subjects and distribution shift.

**Figure 9:**
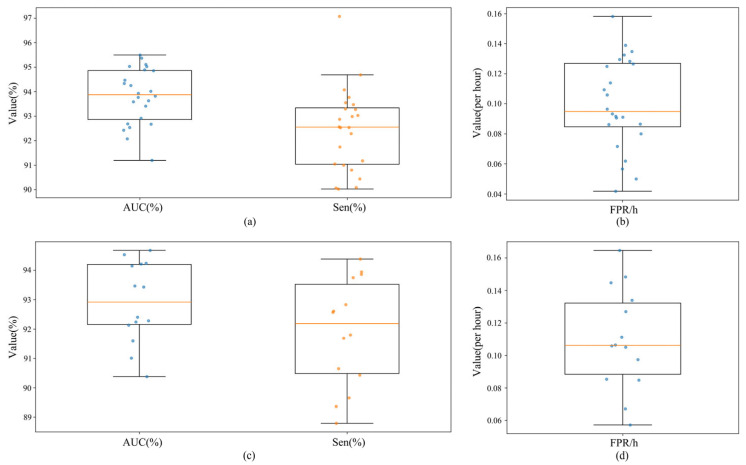
(**a**) CHB-MIT (10-fold cross-subject CV): per-subject AUC & Sen; (**b**) CHB-MIT (10-fold cross-subject CV): per-subject FPR/h; (**c**) Siena (10-fold cross-subject CV): per-subject AUC & Sen; (**d**) Siena (10-fold cross-subject CV): per-subject FPR/h.

Figure 9 presents box plots and scatter plots of subject-level AUC, Sen, and FPR/h on CHB-MIT and Siena, illustrating distributions and stability under cross-subject evaluation. As shown in Figure 9, the subject-level distributions across the four panels align with the mean ± standard deviation in Table 9, indicating stable overall performance under cross-subject evaluation. On CHB-MIT (Figure 9a,b), the medians of AUC and Sen are high, and the boxes are narrow, indicating that most subjects achieve comparable discriminative ability. The median and interquartile width of FPR/h agree with the previously reported 0.10, and the whiskers are short, suggesting controlled inter-subject variability in false alarms.

On Siena (Figure 9c,d), the medians of AUC and Sen are slightly lower than on CHB-MIT, yet the box and whisker ranges remain compact and the dispersion of FPR/h is similar to CHB-MIT, indicating that cross-center differences did not yield extremely unstable cases. Overall, the four plots exhibit high medians, narrow interquartile ranges, and few point outliers, indicating that without sharing subject information, the model maintains consistent discriminative levels across individuals and datasets, which corroborates the mean ± standard deviation in Table 9 and the robustness under cross-subject evaluation. Taken together, the preserved coupling between Acc and Spe under the original class imbalance, along with uniformly small standard deviations across metrics, indicated that the joint design of FiLM modulation, ring buffer memory, and multi-teacher distillation maintained reliable cross-subject and cross-dataset generalization under lightweight constraints.

While Figure 9 illustrates the macro-averaged performance distributions, relying solely on aggregated metrics may mask individual failures, which are critical for clinical safety. To rigorously evaluate the model’s robustness against inter-subject variability and domain shifts, we report the detailed subject-level performance for the cross-dataset experiments in Table 10 and Table 11.

Table 10 details the results of Experiment 3, where the model was trained on the CHB-MIT dataset and tested on Siena patients. Despite the significant differences in recording equipment and electrode montages, the RGF-Model achieved 100% seizure prediction Sen on 50% of the subjects (PN00, PN05, PN12, PN14) with low false alarm rates (FPR < 0.15/h). A performance dip was observed in specific subjects, such as PN09 and PN13, where Sen dropped to 66.67%. This variation contributes to the higher standard deviation observed in the cross-dataset results compared to the within-dataset validation (Exp. 1 and Exp. 2). However, it is noteworthy that even in these challenging cases, the model maintained a detection Acc above 86% and an AUC above 74%, indicating that the model retains discriminative capability and avoids complete failure even under significant distribution shifts.

Similarly, Table 11 presents the subject-level breakdown for Experiment 4 (Training on Siena, Testing on CHB-MIT). The model demonstrated strong generalization capabilities on the majority of the CHB-MIT cohort, achieving 100% prediction Sen on 5 out of 16 patients (Pt1, Pt5, Pt9, Pt14, Pt19) and maintaining an FPR below 0.2/h for most subjects. The relatively lower performance observed in subjects like Pt8 and Pt16 (Sen < 75%, FPR > 0.3/h) can be attributed to their complex seizure patterns and frequent artifacts, which are notoriously difficult to generalize to without subject-specific calibration. Nevertheless, the consistent detection AUC scores (mean 88.70 ± 5.25%) across the cohort suggest that the FiLM-modulated feature extraction mechanism effectively mitigates the negative impact of domain heterogeneity, ensuring a baseline level of reliability for unseen patients.

### 3.5. Robustness Testing of Teacher Model Random Pairing

To test whether random teacher pairing within mini-batches would introduce noticeable variability across runs under the same setting, five independent repetitions of training and evaluation were conducted for the RGF Model (Full KD) on CHB-MIT, and Siena, without changing data splits, hyperparameters, or post-processing, varying only the random seed. Each run used subject-wise LOOCV. For each run, metrics were computed for each subject, then averaged at the subject level to obtain an overall score for that run, and finally summarized across the five runs as mean ± SD, where mean denotes average performance and SD quantifies variability due to the random seed. Table 12 reports mean ± SD for the prediction task AUC, Sen, and FPR/h and for the detection task Acc, Sen, Spe, and AUC. Cross-seed SD was small for all metrics, indicating that random teacher pairing did not introduce appreciable instability and that the model exhibited strong robustness and reproducibility.

### 3.6. k-of-n Voting and Refractory Period Sen Analysis

**Figure 10:**
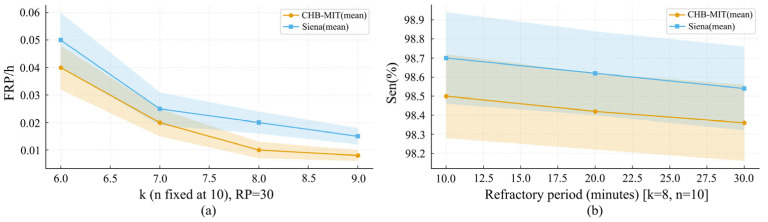
(**a**) shows FPR/h versus k under k of n voting (n fixed at 10, RP = 30 min). The curve denotes the mean, and the shaded region the standard deviation; (**b**) shows Sen as a function of the refractory period with k = 8 and n = 10. The curve denotes the mean, and the shaded region the standard deviation.

To suppress transient noise and improve the temporal stability of alarms, an offline rescan of the voting parameters and the refractory period (RP) was performed while keeping the model outputs fixed. Under a 2 s window and 1s step, n was fixed at 10, k was swept over {6, 7, 8, 9}, and RP was swept over {10 s, 20 s, 30 s}. Metrics were first computed at the subject level and then averaged across subjects to obtain the mean and the standard deviation, which quantified the effect of parameter changes on performance. As shown in Figure 10a, increasing k led to a marked decrease in FPR/h on both CHB-MIT and Siena, whereas Sen declined only mildly. The trends were consistent across datasets, and the standard deviation bands were narrow, indicating that raising the voting threshold suppresses sporadic false triggers without materially compromising detection. With n fixed at 10, k=8 provided a favorable operating point that achieved low FPR/h on both datasets while maintaining high Sen, and matched the response latency implied by a voting window of about 10 s.

As shown in Figure 10b, with k=8 and n=10 fixed, increasing the RP from 10 min to 30 min led to a slight decrease in Sen, while the curves remained smooth with small standard deviations, indicating that a moderate RP extension merges adjacent alarms and suppresses false alarm crosstalk without materially reducing Sen. Based on trends across both datasets, k=8, n=10, and RP = 30 min were adopted as the default configuration, which achieves a robust balance between low FPR/h and high Sen and matches the real time causal inference setting with a 2 s window and 1 s step. Taken together, these results show that the post-processing exhibits consistent effects across datasets and enables reliable false alarm suppression and a performance latency trade-off through simple, tunable temporal gating without changing model parameters.

Crucially, regarding the clinical implications of the resulting response latency, the fixed window size (n=10) introduces a mandatory decision delay of approximately 8–10 s. We acknowledge that this latency is a critical factor; however, in the context of wearable monitoring, it represents a necessary trade-off to strictly minimize the False Positive Rate (FPR) and prevent alarm fatigue caused by transient motion artifacts. From a clinical perspective, this delay remains within the actionable window. For seizure prediction, it is negligible compared to the typical warning horizon (e.g., 30 min). For detection, an 8–10 s lag does not materially hinder the recognition of prolonged emergencies such as status epilepticus, which are clinically defined by durations extending over several minutes (typically >5 min), nor does it preclude timely interventions such as neurostimulation or rescue medication administration.

### 3.7. Comparative Experiments with Other Methods

To systematically evaluate the effectiveness of the RGF-Model for seizure prediction and detection, this section presents a detailed comparison with its teacher models and recent knowledge distillation methods. As shown in Table 13, the RGF-Model exhibits clear advantages over teacher models in both predictive performance and lightweight design. On the CHB-MIT dataset, teacher models such as CBAM-3D CNN-LSTM [39], Gatformer [7], GAT-TCN [6], and Multidimensional Transformer and LSTM-GRU Fusion [8] achieved AUC values of 98.52%, 99.10%, 98.67%, and 99.64%, respectively. Their Sen ranged from 97% to 98.5%, with FPR/h between 0.01 and 0.03.

**Figure 11:**
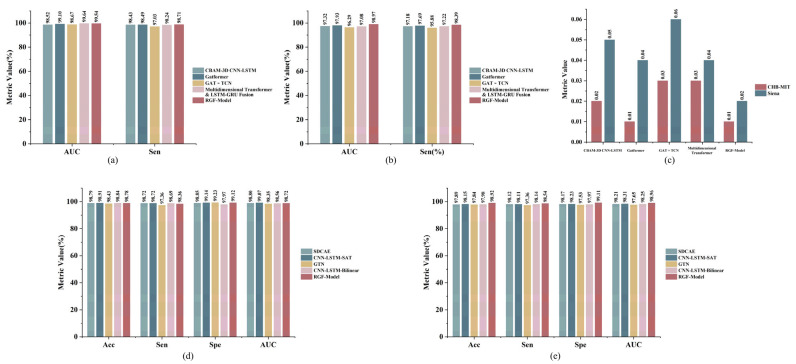
(**a**) Prediction performance of the RGF-Model and teacher models on the CHB-MIT dataset; (**b**) Prediction performance of the RGF-Model and teacher models on the Siena dataset; (**c**) FPR/h of the RGF-Model and teacher models on the CHB-MIT and Siena datasets; (**d**) Detection performance of the RGF-Model and teacher models on the CHB-MIT dataset; (**e**) Detection performance of the RGF-Model and teacher models on the Siena dataset.

**Figure 12:**
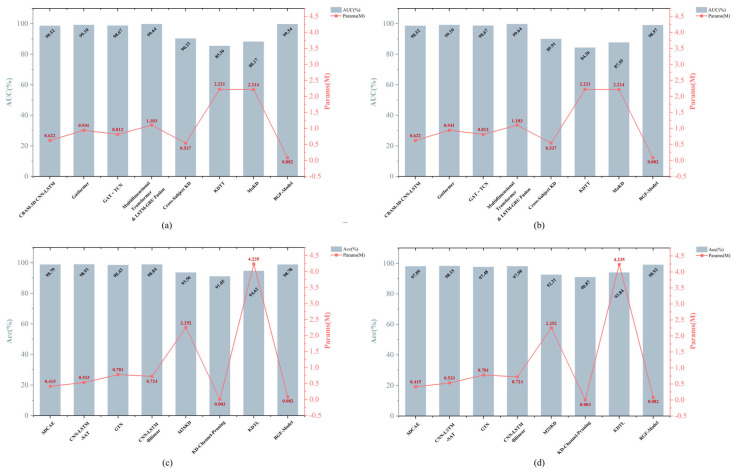
(**a**) Performance and parameter scale of the RGF-Model and other prediction models on the CHB-MIT dataset; (**b**) Performance and parameter scale of the RGF-Model and other prediction models on the Siena dataset; (**c**) Detection performance and parameter scale of the RGF-Model and other detection models on the CHB-MIT dataset; (**d**) Detection performance and parameter scale of the RGF-Model and other detection models on the Siena dataset.

By contrast, under the multi-teacher knowledge distillation strategy, the RGF-Model achieved an AUC of 99.54%, a Sen of 98.71%, and an FPR/h of 0.01. The performance comparison is illustrated in Figure 11. On the Siena dataset, the results summarized in Table 14 show that teacher models achieved AUC values mostly between 96% and 98%. The RGF-Model, however, reached an AUC of 98.75%, a Sen of 98.55%, and a low FPR/h of 0.02, demonstrating strong generalization capability across datasets. In terms of complexity, teacher models typically contain 0.6–1.1 M parameters and occupy 2–4.4 MB of storage. In contrast, the student model requires only 0.06–0.09 M parameters, with model sizes of 0.24–0.36 MB. The comparison of model performance and parameter scale is illustrated in Figure 12.

In the seizure detection task, the RGF-Model demonstrated strong performance. On the CHB-MIT dataset, as shown in Table 15, teacher models including SDCAE [9], CNN-LSTM-SAT [40], GTN [41], and CNN-LSTM-Bilinear [10] achieved Acc of 98.79%, 98.91%, 98.43%, and 98.84%, respectively, with Sen and Spe between 97% and 99%. In contrast, the optimal RGF-Model combination slightly outperformed the teacher models. Moreover, the teacher models in the detection task required relatively large parameter counts, such as 0.41 M for SDCAE-Bi-LSTM and 0.72 M for Hybrid Bilinear CNN-LSTM, whereas the RGF-Model contained only 0.07 M parameters with storage below 0.3 MB. These results indicated that the RGF-Model achieved a favorable balance between compactness, detection Acc, and generalization, thereby enhancing its deployability in low-resource real-time scenarios. Furthermore, a consistent trend was observed on the Siena dataset. As shown in Table 16, the teacher models SDCAE [9], CNN-LSTM-SAT [40], GTN [41], and CNN-LSTM-Bilinear [10] achieved Acc of 97.89%, 98.15%, 97.84%, and 97.98%, with Sen between 97% and 99%, Spe between 97% and 99%, and AUC between 98% and 99%. Under this baseline, the lightweight RGF-Model, with about 0.082 M parameters and a 0.33 MB model size, achieved Acc 98.92%, Sen 98.54%, Spe 99.11%, and AUC 98.96%, yielding performance comparable to or better than the larger teacher models and further confirming that the multi-teacher distillation framework maintains high detection Acc while substantially reducing model complexity.

It is particularly noteworthy that the distilled RGF-Model outperformed its teacher models in several key metrics, contradicting the common assumption that student models are merely degraded approximations. Specifically, on the Siena dataset, the student surpassed the teacher models by 1.89% in AUC and 1.17% in Sen for prediction, while significantly reducing the FPR/h to 0.02 (compared to 0.04–0.06 for teachers). This performance gain can be attributed to three factors: First, the multi-teacher framework creates an ensemble effect, where the student integrates complementary global dependencies from the prediction teacher and local transient features from the detection teacher, acquiring a more comprehensive view than any single teacher. Second, the structural advantages of the student model play a critical role; the FiLM-modulated Ring-GRU mechanism provides superior continuous state tracking and dynamic task adaptation compared to the standard RNNs or Transformers used in the teachers, directly contributing to the suppression of false alarms. Third, knowledge distillation serves as a strong regularizer. Large teacher models (e.g., >1 M parameters) are prone to overfitting on smaller datasets like Siena, whereas the student, learning from smoothed soft labels, captures more robust data distributions, thereby achieving better generalization on unseen data.

**Figure 13:**
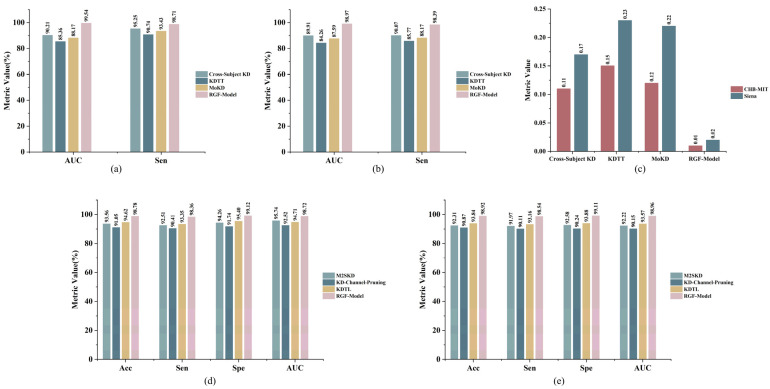
(**a**) Prediction performance of the RGF-Model and existing KD models on the CHB-MIT dataset; (**b**) Prediction performance of the RGF-Model and existing KD models on the Siena dataset; (**c**) FPR/h of the RGF-Model and existing KD models on the CHB-MIT and Siena; (**d**) Detection performance of the RGF-Model and existing KD models on the CHB-MIT dataset; (**e**) Detection performance of the RGF-Model and existing KD models on the Siena dataset.

This study also conducted a comparative analysis of recent lightweight knowledge distillation models for epilepsy prediction and detection. For the prediction task, as shown in Table 17, although Cross-Subject KD [19], KDTT [51], and MoKD [18] improved performance through cross-subject or cross-modal distillation, their AUC on CHB-MIT remained between 85% and 90%, with Sen between 90% and 95%, and FPR/h between 0.11 and 0.15. Their parameter counts were 0.53 M, 2.22 M, and 2.21 M, respectively, corresponding to model sizes from 2 MB to 8.85 MB. On the Siena dataset, performance further deteriorated, with KDTT and MoKD achieving AUC 84.26% and 87.59%, and FPR/h 0.23 and 0.22. In contrast, the RGF-Model achieved AUC and Sen above 98% on the same dataset, with FPR/h below 0.02, significantly outperforming these methods. Model performance comparisons are presented in Figure 13. Additionally, as shown in Figure 12, the RGF-Model contained significantly fewer parameters, only one-tenth or less than other knowledge distillation models, while maintaining superior performance. These results demonstrated that the FiLM causal gating and SFPM effectively enhanced temporal causal consistency and generalization, making the RGF-Model more suitable for real-time epilepsy prediction than existing knowledge distillation strategies.

In epilepsy detection tasks, although methods such as M2SKD [18], KD-Channel-Pruning [52], and KDTL [53] had achieved progress, a clear trade-off between performance and model size remained. As shown in Table 18, although M2SKD and KDTL achieved good detection performance, their parameter counts (2.252 M and 4.235 M) and storage requirements (8.77 MB and 16.85 MB) severely limited deployment on low-resource platforms. KD-Channel-Pruning, while extremely compact, suffered from oversimplification, yielding Acc 91.05% and 90.87%, which was inadequate. In contrast, the proposed RGF-Model achieved Acc, Sen, and AUC above 98% with only 0.07 M parameters, outperforming KD-Channel-Pruning and providing more than tenfold compression compared with M2SKD and KDTL. This substantially reduced computational cost and enhanced suitability for real-time deployment.

Finally, a comprehensive comparison was conducted between the proposed RGF-Model and recent mainstream deep learning methods, including GGN [54], AMSRN [55], and the lightweight LightSeizureNet [56]. Although AMSRN and GGN showed strong feature extraction capability (e.g., GGN achieved detection AUC above 98%), their multi-million parameter counts hindered deployment in resource-constrained scenarios. LightSeizureNet achieved efficient inference via pruning, but was limited to detection tasks and unsuitable for prediction, which reduced its generalization and application scope. In contrast, the RGF-Model integrated real-time prediction and detection. Through knowledge distillation and causal gating, it achieved an order of magnitude reduction in parameters while preserving high performance. Moreover, it ensured temporal consistency and real-time responsiveness, enhancing practicality and versatility.

In summary, comparisons with mainstream teacher models and distillation methods showed that the proposed RGF-Model achieved consistent improvements in prediction and detection performance, model compactness, cross-dataset generalization, and causal consistency. With minimal computational demand and efficient task handling, the RGF-Model demonstrated strong theoretical potential and practical value for real-time epilepsy monitoring in resource-constrained settings.

### 3.8. On-Device Efficiency Benchmarks

To strengthen the engineering feasibility of the lightweight argument and avoid relying solely on parameter count and model size, the student model’s on-device efficiency was systematically evaluated under a unified and reproducible benchmark. All tests were run on a general-purpose CPU using single-threaded streaming inference with a batch size of 1, a 2s window, and a 1s step. End-to-end latency covered the full path from input to output, including preprocessing and post-processing. To ensure representativeness and comparability, on the prediction side, the multidimensional Transformer and LSTM-GRU fusion [8] and Gatformer [7] served as teachers, and CBAM-3D CNN-LSTM [39] and GAT-TCN [6] were included as representative methods. On the detection side, CNN-LSTM-SAT [40] and SDCAE [9] served as teachers, and GTN [41] and CNN-LSTM-Bilinear [10] were used as representative methods. All models were strong baselines used in the main text and covered diverse inductive biases, including convolutional, recurrent, attention-based, graph-based, and bilinear designs. For fair comparison, automatic multithreading and asynchronous execution were disabled during testing. The student, teacher, and representative models shared identical input channels, time chunking strategy, montage selection, and referencing scheme, and ran on the same software stack. Detailed hardware and software settings are listed in Section 2.8.

This section reports three key metrics relevant to edge deployment. The first category was computation and storage cost, including parameter count, model file size, FLOPs per 2s window, and peak memory usage during inference. FLOPs were computed by exact analytical counting over convolutional and fully connected operators under a unified standard. Memory usage was measured as the peak allocation observed during streaming inference. The second category was latency cost, covering end-to-end inference time per 2s window and the real-time factor, defined as the ratio of inference latency to 2000 ms. All latency values were reported as the mean and the standard deviation over multiple runs to reflect stability and variability. The third category was energy cost, estimated from FLOPs and a per-operation energy coefficient from the literature, yielding the per inference energy order of magnitude. This estimate provided a scale reference rather than measured power on wearable devices. The same coefficient was applied to all methods to ensure fair comparison.

Under the unified setting above, a comparative table covering four dimensions—resource usage, computational complexity, inference latency, and energy—was provided, listing side-by-side results for the student, teacher, and representative models. The table complemented the main performance results and demonstrated that the student model preserved high Acc while offering significant on-device efficiency. Under a unified CPU baseline, end-to-end efficiency metrics, including latency and energy estimates, were reported, as shown in Table 19, and the results indicated that the student model achieved clear advantages in resource usage and inference time. Future work will include latency and energy measurements on real edge devices and a systematic comparison with teacher and representative models to validate deployment performance and practical applicability.

To systematically evaluate the effectiveness of the RGF-Model for seizure prediction and detection, this section presents a detailed comparison with its teacher models and recent knowledge distillation methods.

## 4. Discussion

The RGF-Model addresses the critical trade-off between computational efficiency and high-dimensional feature extraction required for wearable epilepsy monitoring. Unlike traditional deep learning architectures that rely on parameter redundancy to ensure performance [11,14], our design reconciles these conflicting demands through the integration of Ring-GRU and FiLM. The Ring-GRU mechanism overcomes the memory decay inherent in standard RNNs by enforcing a fixed-budget causal memory, ensuring that long-range temporal dependencies are captured without violating real-time constraints [4]. Simultaneously, the prioritization of FiLM validated a superior gain-to-cost strategy, enabling the shared backbone to adaptively separate task-specific representations with minimal overhead compared to computationally intensive attention stacking.

Beyond structural efficiency, the multi-teacher knowledge distillation strategy functions as a critical regularization mechanism. While single-teacher methods often struggle with task conflict or domain overfitting [13,16,17], our framework integrates complementary inductive biases—synthesizing global temporal contexts from the Transformer teacher with local transient patterns from the CNN-LSTM teacher. This synergistic guidance allows the lightweight student to approximate the complex decision boundaries of teacher ensembles [17,52]. Consequently, the model demonstrates robust generalization on unseen data, confirming that distilling knowledge from diverse architectures effectively mitigates the overfitting often observed when training on limited EEG datasets.

Furthermore, the unification of prediction and detection within a single causal framework resolves the reliability issues inherent in independent modeling [4,6]. By utilizing the detection output as a dynamic SFPM, the system strictly prevents look-ahead bias while enhancing Sen to subtle preictal shifts [30,57]. This low-latency, causally consistent paradigm holds significant potential for broader biosignal applications requiring rapid decoding, such as movement imagination-based remote vehicle control [58], where immediate response is paramount for user safety.

Despite these contributions, a primary limitation stems from the post-processing strategy designed to minimize false alarms. The Ring-Buffer mechanism enforces a refractory period to prevent alarm fatigue, which fundamentally trades off the detection of acute seizure clusters for lower false positive rates. While this design prioritizes user compliance in wearable settings by treating a cluster as a single actionable event, it may mask subsequent seizures occurring within the suppression window. Future work will address this by exploring adaptive refractory mechanisms to distinguish between prolonged artifacts and genuine repetitive seizures, alongside investigating multimodal fusion to further enhance clinical robustness.

## 5. Conclusions

This study proposed the RGF-Model based on multi-teacher knowledge distillation for efficient and accurate early prediction and detection of epileptic seizures. The model integrated Ring-GRU and FiLM, and the Ring-GRU structure ensured strict causality by relying solely on historical and current information. The FiLM mechanism enabled task-specific dynamic modulation of shared features, thereby enhancing synergy between prediction and detection. Furthermore, a multi-teacher knowledge distillation strategy was employed to guide the student to learn task-specific knowledge from both tasks, which substantially reduced parameter count and computational complexity while maintaining high performance.

The experimental results showed that, by adopting the structurally complementary and high performing Multidimensional Transformer (prediction) and CNN-LSTM with Attention (detection) as teacher models and combining them with a multi-teacher knowledge distillation strategy, the RGF-Model achieved prediction AUCs of 99.54% and 98.79% with FPR/h of 0.01 and 0.02, and detection Acc of 98.78% and 98.92% on CHB-MIT and Siena, respectively. In addition, the model contained about 0.07 M parameters, which was an order of magnitude fewer than existing methods, demonstrating strong generalization and stability.

The Ring-GRU architecture efficiently captured long-range temporal dependencies via ring-shaped gated updates, mitigating the memory decay problem of traditional recurrent neural networks. The FiLM mechanism adaptively modulated task-specific features while adding minimal parameters. The multi-teacher knowledge distillation strategy enabled efficient knowledge transfer, allowing the student model to achieve strong performance under limited computational resources. This design reduced model complexity and highlighted its potential for real-time deployment on low-power embedded and wearable devices. Furthermore, comparative experiments with other deep learning methods validated the model’s significant advantages in Acc, generalization, and computational efficiency. Cross-dataset experiments further verified the model’s strong generalization. In addition, ablation experiments also confirmed the effectiveness of each module.

In summary, the RGF-Model proposed in this study provided an efficient, lightweight, and accurate framework for epileptic seizure prediction and detection and offered a clear path and starting point for subsequent validation on wearable devices and clinical translation. Because of hardware constraints, this work did not include real power measurements on wearable devices. Future work will further explore personalized training and multimodal data fusion and will add on-device measurements of latency and power to enhance generalization and clinical applicability.

## Figures and Tables

**Table 1:** Summary of the CHB-MIT dataset used in this work.

No. of Patients	Interictal Hours (h)	No. of Seizures
Pt1	17.7	7
Pt2	23.1	3
Pt3	21.9	6
Pt5	14.4	5
Pt8	3.5	5
Pt9	50	4
Pt10	26	6
Pt13	15.6	5
Pt14	4.2	5
Pt16	5.6	10
Pt17	10.1	3
Pt18	25	6
Pt19	23.1	3
Pt20	20.8	5
Pt21	21.6	4
Pt23	14.2	5
Total	296.8	82

**Table 2:** Summary of the Siena dataset used in this work.

No. of Patients	Interictal Hours (h)	No. of Seizures
PN00	3.2	5
PN05	6	3
PN06	12	5
PN09	6.8	3
PN10	16.6	10
PN12	4	4
PN13	5.6	3
PN14	23.4	4
Total	77.6	37

**Table 3:** CHB-MIT Multi-Teacher Combination Distillation RGF-Model Performance on Prediction Tasks.

Prediction Methods	Detection Methods	AUC (%)	Sen (%)	FPR/h	Sig. (%)	Params (M)	Model Size (MB)
CBAM-3D CNN -LSTM [39]	SDCAE [9]	98.40 ± 1.42	98.21 ± 2.02	0.03 ± 0.015	100%	0.060	0.24
	CNN-LSTM-SAT [40]	98.33 ± 1.77	97.94 ± 1.93	0.02 ± 0.014	100%	0.064	0.26
	GTN [41]	98.71 ± 1.64	98.45 ± 1.79	0.01 ± 0.005	100%	0.073	0.29
	CNN-LSTM-Bilinear [10]	98.55 ± 1.56	98.28 ± 2.11	0.02 ± 0.009	100%	0.068	0.27
Gatformer [7]	SDCAE [9]	98.46 ± 1.29	97.85 ± 1.64	0.02 ± 0.013	100%	0.071	0.28
	CNN-LSTM-SAT [40]	98.77 ± 1.29	98.50 ± 1.79	0.01 ± 0.005	100%	0.076	0.30
	GTN [41]	99.02 ± 1.23	98.55 ± 1.87	0.01 ± 0.004	100%	0.084	0.34
	CNN-LSTM-Bilinear [10]	98.88 ± 1.72	98.43 ± 1.96	0.01 ± 0.005	100%	0.081	0.32
GAT-TCN [6]	SDCAE [9]	97.95 ± 1.56	96.81 ± 2.29	0.04 ± 0.016	100%	0.066	0.26
	CNN-LSTM-SAT [40]	98.22 ± 1.62	97.40 ± 1.70	0.03 ± 0.014	100%	0.072	0.28
	GTN [41]	97.68 ± 1.21	96.55 ± 2.01	0.05 ± 0.019	100%	0.078	0.31
	CNN-LSTM-Bilinear [10]	98.11 ± 1.78	97.02 ± 2.09	0.04 ± 0.025	100%	0.074	0.30
Multidimensional Transformer & LSTM-GRU Fusion [8]	SDCAE [9]	99.31 ± 1.70	98.62 ± 1.55	0.02 ± 0.010	100%	0.079	0.32
	CNN-LSTM-SAT [40]	99.54 ± 1.33	98.71 ± 2.11	0.01 ± 0.006	100%	0.082	0.33
	GTN [41]	99.12 ± 1.31	98.37 ± 1.67	0.02 ± 0.012	100%	0.091	0.36
	CNN-LSTM-Bilinear [10]	99.43 ± 1.31	98.59 ± 1.57	0.01 ± 0.004	100%	0.086	0.34
	Aver	98.66	98.02	0.02	-	0.075	0.30

**Table 4:** Siena Multi-Teacher Combination Distillation RGF-Model Performance on Prediction Tasks.

Prediction Methods	Detection Methods	AUC (%)	Sen (%)	FPR/h	Sig. (%)	Params (M)	Model Size (MB)
CBAM-3D CNN -LSTM [39]	SDCAE [9]	97.12 ± 1.65	96.83 ± 2.24	0.05 ± 0.035	100%	0.060	0.24
	CNN-LSTM-SAT [40]	97.30 ± 2.29	97.74 ± 1.91	0.03 ± 0.021	100%	0.064	0.26
	GTN [41]	96.95 ± 1.67	96.43 ± 2.98	0.02 ± 0.014	100%	0.073	0.29
	CNN-LSTM-Bilinear [10]	97.25 ± 1.81	96.98 ± 2.99	0.05 ± 0.028	100%	0.068	0.27
Gatformer [7]	SDCAE [9]	97.47 ± 1.93	97.18 ± 3.16	0.04 ± 0.013	100%	0.071	0.28
	CNN-LSTM-SAT [40]	97.68 ± 2.62	97.75 ± 2.34	0.03 ± 0.014	100%	0.076	0.31
	GTN [41]	98.21 ± 2.37	97.93 ± 3.14	0.04 ± 0.022	100%	0.084	0.34
	CNN-LSTM-Bilinear [10]	97.45 ± 2.20	97.54 ± 2.42	0.02 ± 0.011	100%	0.081	0.32
GAT-TCN [6]	SDCAE [9]	96.95 ± 1.54	96.62 ± 2.74	0.06 ± 0.033	100%	0.066	0.26
	CNN-LSTM-SAT [40]	97.11 ± 2.45	96.81 ± 1.92	0.04 ± 0.019	100%	0.072	0.28
	GTN [41]	96.76 ± 2.01	96.38 ± 3.06	0.05 ± 0.029	100%	0.078	0.31
	CNN-LSTM-Bilinear [10]	97.09 ± 2.54	96.77 ± 2.22	0.05 ± 0.024	100%	0.074	0.30
Multidimensional Transformer & LSTM-GRU Fusion [8]	SDCAE [9]	98.03 ± 1.83	97.97 ± 2.17	0.04 ± 0.021	100%	0.079	0.32
	CNN-LSTM-SAT [40]	98.97 ± 1.57	98.39 ± 1.81	0.02 ± 0.009	100%	0.082	0.33
	GTN [41]	98.12 ± 2.63	97.87 ± 2.56	0.04 ± 0.016	100%	0.091	0.35
	CNN-LSTM-Bilinear [10]	98.43 ± 1.79	98.29 ± 2.47	0.02 ± 0.015	100%	0.086	0.34
	Aver	97.56	97.34	0.04	-	0.075	0.30

**Table 5:** CHB-MIT Multi-Teacher Combination Distillation RGF-Model Performance on detection Tasks.

Prediction Methods	Detection Methods	Acc (%)	Sen (%)	Spe (%)	AUC (%)	Params (M)	Model Size (MB)
CBAM-3D CNN -LSTM [39]	SDCAE [9]	98.34 ± 1.20	98.36 ± 1.43	98.33 ± 1.46	98.82 ± 0.97	0.060	0.24
	CNN-LSTM -SAT [40]	98.33 ± 1.04	98.14 ± 1.30	98.52 ± 0.84	98.32 ± 1.18	0.064	0.26
	GTN [41]	98.01 ± 1.05	97.41 ± 1.47	98.65 ± 1.01	98.23 ± 1.05	0.073	0.29
	CNN-LSTM -Bilinear [10]	98.46 ± 1.14	98.27 ± 1.20	98.70 ± 0.99	98.56 ± 1.10	0.068	0.27
Gatformer [7]	SDCAE [9]	98.57 ± 0.83	98.22 ± 1.21	98.91 ± 0.96	98.54 ± 0.71	0.071	0.28
	CNN-LSTM -SAT [40]	98.74 ± 1.20	98.61 ± 1.16	98.94 ± 0.73	98.91 ± 0.74	0.076	0.30
	GTN [41]	98.62 ± 0.84	98.05 ± 1.24	99.20 ± 1.01	98.75 ± 1.00	0.084	0.34
	CNN-LSTM -Bilinear [10]	98.69 ± 1.06	98.33 ± 1.30	99.02 ± 1.13	98.80 ± 0.89	0.081	0.32
GAT-TCN [6]	SDCAE [9]	97.88 ± 1.73	97.54 ± 1.24	98.21 ± 1.38	97.85 ± 1.25	0.066	0.26
	CNN-LSTM -SAT [40]	98.11 ± 1.09	97.72 ± 1.43	98.43 ± 1.16	98.15 ± 0.98	0.072	0.28
	GTN [41]	97.36 ± 1.44	96.84 ± 1.24	98.25 ± 1.45	97.35 ± 1.28	0.078	0.31
	CNN-LSTM -Bilinear [10]	97.75 ± 1.45	97.19 ± 1.33	98.03 ± 1.37	97.56 ± 1.53	0.074	0.30
Multidimensional Transformer & LSTM-GRU Fusion [8]	SDCAE [9]	98.72 ± 0.69	98.64 ± 1.16	98.80 ± 1.04	98.90 ± 0.92	0.079	0.32
	CNN-LSTM -SAT [40]	98.78 ± 1.18	98.36 ± 1.46	99.12 ± 0.67	98.72 ± 1.17	0.082	0.33
	GTN [41]	98.56 ± 1.15	98.49 ± 1.33	98.68 ± 1.15	98.85 ± 0.95	0.091	0.36
	CNN-LSTM -Bilinear [10]	98.67 ± 0.94	98.41 ± 1.34	98.93 ± 0.83	98.95 ± 0.92	0.086	0.34
	Aver	98.35	98.04	98.67	98.45	0.075	0.30

**Table 6:** Siena Multi-Teacher Combination Distillation RGF-Model Performance on detection Tasks.

Prediction Methods	Detection Methods	Acc (%)	Sen (%)	Spe (%)	AUC (%)	Params (M)	Model Size (MB)
CBAM-3D CNN -LSTM [39]	SDCAE [9]	98.63 ± 1.08	98.18 ± 1.33	98.95 ± 0.71	98.62 ± 1.37	0.060	0.24
	CNN-LSTM -SAT [40]	98.68 ± 1.01	98.26 ± 1.50	98.82 ± 0.82	98.73 ± 1.46	0.064	0.26
	GTN [41]	98.72 ± 1.09	98.33 ± 1.58	98.76 ± 0.91	98.74 ± 1.40	0.073	0.29
	CNN-LSTM -Bilinear [10]	98.74 ± 1.30	98.32 ± 1.58	98.88 ± 0.97	98.76 ± 1.48	0.068	0.27
Gatformer [7]	SDCAE [9]	98.70 ± 1.24	98.31 ± 1.25	98.71 ± 1.29	98.72 ± 1.23	0.071	0.28
	CNN-LSTM -SAT [40]	98.78 ± 1.14	98.38 ± 1.55	98.98 ± 1.13	98.84 ± 0.72	0.076	0.30
	GTN [41]	98.62 ± 1.13	98.42 ± 1.70	98.63 ± 1.05	98.84 ± 0.79	0.084	0.34
	CNN-LSTM -Bilinear [10]	98.84 ± 0.71	98.44 ± 1.38	98.85 ± 1.16	98.86 ± 0.98	0.081	0.32
GAT-TCN [6]	SDCAE [9]	98.62 ± 1.00	98.22 ± 1.79	98.97 ± 1.28	98.64 ± 1.31	0.066	0.26
	CNN-LSTM -SAT [40]	98.71 ± 0.97	98.31 ± 1.53	98.75 ± 1.22	98.73 ± 1.31	0.072	0.28
	GTN [41]	98.75 ± 1.32	98.35 ± 1.78	98.89 ± 0.96	98.77 ± 1.34	0.078	0.31
	CNN-LSTM -Bilinear [10]	98.78 ± 1.42	98.38 ± 1.41	98.82 ± 1.00	98.81 ± 1.29	0.074	0.30
Multidimensional Transformer & LSTM-GRU Fusion [8]	SDCAE [9]	98.80 ± 1.10	98.42 ± 1.51	98.93 ± 1.16	98.84 ± 0.94	0.079	0.32
	CNN-LSTM -SAT [40]	98.92 ± 1.30	98.54 ± 1.49	99.11 ± 0.89	98.96 ± 1.01	0.082	0.33
	GTN [41]	98.86 ± 0.78	98.48 ± 1.32	99.08 ± 0.80	98.90 ± 1.25	0.091	0.36
	CNN-LSTM -Bilinear [10]	98.91 ± 1.00	98.52 ± 1.49	99.03 ± 1.07	98.94 ± 0.93	0.086	0.34
	Aver	98.75	98.37	98.89	98.79	0.075	0.30

**Table 7:** CHB-MIT Epilepsy Prediction and Detection Performance Ablation Experiment Results.

Ablation	Seizure Prediction				Seizure Detection				Params (M)	Model Size (MB)
	AUC (%)	Sen (%)	FPR/h	Sig. (%)	Acc (%)	Sen (%)	Spe (%)	AUC (%)		
Full KD	99.54 ± 1.36	98.71 ± 1.50	0.01 ± 0.006	100%	98.78 ± 1.31	98.36 ± 1.28	99.12 ± 1.40	98.72 ± 1.78	0.082	0.33
No FiLM	96.42 ± 2.60	96.73 ± 2.26	0.03 ± 0.014	100%	96.52 ± 2.05	96.21 ± 1.90	97.02 ± 2.26	96.88 ± 2.19	0.079	0.31
No Transformer	97.27 ± 1.91	96.83 ± 2.09	0.04 ± 0.022	100%	97.23 ± 2.01	97.30 ± 2.05	96.73 ± 2.44	96.79 ± 2.19	0.075	0.30
No SFPM	97.12 ± 2.13	98.03 ± 1.36	0.03 ± 0.014	100%	98.55 ± 1.28	98.29 ± 1.60	97.98 ± 1.63	98.05 ± 1.32	0.081	0.31
No TimeReg	98.26 ± 1.39	98.11 ± 1.55	0.03 ± 0.014	100%	97.69 ± 1.71	98.19 ± 1.41	97.77 ± 1.61	98.08 ± 1.75	0.081	0.31
Only prediction teacher	98.92 ± 1.29	98.30 ± 1.39	0.02 ± 0.014	100%	-	-	-	-	0.082	0.33
Only detection teacher	-	-	-	-	98.45 ± 1.79	97.98 ± 1.84	98.95 ± 1.36	98.36 ± 1.71	0.082	0.33
No KD	95.78 ± 3.13	95.21 ± 3.41	0.07 ± 0.035	100%	96.12 ± 2.78	95.58 ± 3.23	96.84 ± 2.29	96.10 ± 2.04	0.082	0.33

**Table 8:** Siena Epilepsy Prediction and Detection Performance Ablation Experiment Results.

Ablation	Seizure Prediction				Seizure Detection				Params (M)	Model Size (MB)
	AUC (%)	Sen (%)	FPR/h	Sig. (%)	Acc (%)	Sen (%)	Spe (%)	AUC (%)		
Full KD	98.97 ± 1.51	98.39 ± 2.01	0.02 ± 0.013	100%	98.92 ± 1.69	98.54 ± 1.49	99.11 ± 1.57	98.96 ± 1.77	0.082	0.33
No FiLM	97.03 ± 2.51	96.95 ± 2.65	0.02 ± 0.010	100%	96.87 ± 2.85	96.57 ± 3.30	97.13 ± 2.37	97.05 ± 2.73	0.079	0.31
No Transformer	97.54 ± 2.02	97.02 ± 2.60	0.02 ± 0.014	100%	97.17 ± 2.45	96.93 ± 2.99	97.26 ± 1.85	96.88 ± 2.46	0.075	0.30
No SFPM	96.97 ± 2.94	97.11 ± 1.86	0.04 ± 0.019	100%	98.21 ± 1.81	98.18 ± 2.58	98.27 ± 2.18	97.94 ± 2.46	0.081	0.31
No TimeReg	97.77 ± 2.53	97.61 ± 2.03	0.03 ± 0.024	100%	97.05 ± 2.33	96.98 ± 2.86	97.24 ± 1.99	97.16 ± 1.87	0.081	0.31
Only prediction teacher	98.22 ± 2.35	98.30 ± 2.23	0.02 ± 0.008	100%	-	-	-	-	0.082	0.33
Only detection teacher	-	-	-	-	98.40 ± 2.06	98.05 ± 2.16	98.83 ± 1.26	98.52 ± 1.56	0.082	0.33
No KD	95.89 ± 3.68	95.47 ± 3.54	0.07 ± 0.038	100%	96.05 ± 3.03	95.42 ± 4.34	96.92 ± 2.82	96.18 ± 2.84	0.082	0.33

**Table 9:** Cross-subject and cross-dataset results.

Experiment	Seizure Prediction				Seizure Detection			
	AUC (%)	Sen (%)	FPR/h	Sig. (%)	Acc (%)	Sen (%)	Spe (%)	AUC (%)
Exp. 1	93.81 ± 1.12	92.43 ± 1.65	0.10 ± 0.03	100%	94.86 ± 0.87	93.52 ± 1.35	94.95 ± 0.93	94.67 ± 1.08
Exp. 2	92.91 ± 1.33	91.88 ± 1.78	0.11 ± 0.03	100%	94.23 ± 0.93	93.15 ± 1.42	94.31 ± 0.94	94.17 ± 1.15
Exp. 3	87.01 ± 8.51	87.92 ± 13.9	0.18 ± 0.09	100%	91.06 ± 3.29	87.87 ± 7.14	91.35 ± 2.89	90.22 ± 5.19
Exp. 4	83.82 ± 8.10	83.02 ± 14.5	0.20 ± 0.08	100%	89.09 ± 3.54	86.42 ± 6.64	88.69 ± 3.21	87.63 ± 5.09
Exp. 5	95.09 ± 1.07	93.92 ± 1.43	0.09 ± 0.02	100%	95.71 ± 0.77	94.65 ± 1.11	95.73 ± 0.87	95.42 ± 0.94

**Table 10:** Subject-level performance for Experiment 3 (Training on CHB-MIT, Testing on Siena).

Patient ID	Seizure Prediction			Seizure Detection			
	AUC (%)	Sen (%)	FPR/h	Acc (%)	Sen (%)	Spe (%)	AUC (%)
PN00	88.43	100.00	0.14	92.13	85.34	92.49	91.19
PN05	96.17	100.00	0.07	94.49	96.19	94.11	95.79
PN06	85.32	80.00	0.21	89.21	82.51	90.14	88.46
PN09	76.54	66.67	0.28	87.43	78.59	88.51	84.19
PN10	92.13	90.00	0.12	93.61	92.39	93.79	93.49
PN12	97.87	100.00	0.09	95.12	98.12	94.81	96.61
PN13	74.31	66.67	0.31	86.29	81.49	86.79	82.14
PN14	85.34	100.00	0.22	90.24	88.31	90.16	89.87
Mean ± SD	87.01 ± 8.51	87.92 ± 13.9	0.18 ± 0.09	91.06 ± 3.29	87.87 ± 7.14	91.35 ± 2.89	90.22 ± 5.19

**Table 11:** Subject-level performance for Experiment 4 (Training on Siena, Testing on CHB-MIT).

Patient ID	Seizure Prediction			Seizure Detection			
	AUC (%)	Sen (%)	FPR/h	Acc (%)	Sen (%)	Spe (%)	AUC (%)
Pt1	94.49	100.00	0.06	94.19	96.49	94.11	95.79
Pt2	76.41	66.67	0.24	86.41	83.11	86.59	84.49
Pt3	86.19	83.33	0.18	89.49	88.19	89.79	89.19
Pt5	93.11	100.00	0.11	92.81	93.59	92.51	93.39
Pt8	69.51	60.00	0.34	83.59	76.39	84.19	80.11
Pt9	92.29	100.00	0.12	91.21	92.81	91.11	91.49
Pt10	83.59	83.33	0.19	88.09	86.49	88.39	87.19
Pt13	82.19	80.00	0.18	89.39	87.19	89.59	88.79
Pt14	95.39	100.00	0.08	93.49	94.81	93.19	94.41
Pt16	73.79	70.00	0.32	82.79	73.49	83.89	78.49
Pt17	74.21	66.67	0.28	85.19	80.41	85.49	83.19
Pt18	84.49	83.33	0.22	87.49	86.11	87.79	86.59
Pt19	91.39	100.00	0.11	91.81	92.49	91.61	92.19
Pt20	80.11	80.00	0.24	86.89	84.21	87.11	85.39
Pt21	77.49	75.00	0.26	84.49	80.31	85.19	82.49
Pt23	86.43	80.00	0.19	88.11	86.59	88.49	88.89
Mean ± SD	83.82 ± 8.10	83.02 ± 14.5	0.20 ± 0.08	89.09 ± 3.54	86.42 ± 6.64	88.69 ± 3.21	87.63 ± 5.09

**Table 12:** Seed Robustness on CHB-MIT & Siena (*n* = 5): Mean ± SD.

Datasets	Seizure Prediction			Seizure Detection			
	AUC (%)	Sen (%)	FPR/h	Acc (%)	Sen (%)	Spe (%)	AUC (%)
CHB-MIT	99.54 ± 0.06	98.71 ± 0.12	0.01 ± 0.003	98.78 ± 0.08	98.36 ± 0.14	99.12 ± 0.06	98.72 ± 0.0.9
Siena	98.97 ± 0.08	98.39 ± 0.13	0.02 ± 0.004	98.92 ± 0.07	98.54 ± 0.12	99.11 ± 0.06	98.96 ± 0.08

**Table 13:** CHB-MIT Teacher Model Epileptic Seizure Prediction Results.

Prediction Methods	AUC (%)	Sen (%)	FPR/h	Sig. (%)	Params (M)	Model Size (MB)
CBAM-3D CNN-LSTM [39]	98.52 ± 1.49	98.43 ± 1.94	0.02 ± 0.016	100%	0.622	2.48
Gatformer [7]	99.10 ± 1.93	98.49 ± 1.95	0.01 ± 0.005	100%	0.941	3.76
GAT-TCN [6]	98.67 ± 1.70	97.03 ± 2.46	0.03 ± 0.016	100%	0.812	3.24
Multidimensional Transformer & LSTM-GRU Fusion [8]	99.64 ± 1.89	98.24 ± 1.91	0.03 ± 0.016	100%	1.103	4.40
RGF-Model	99.54 ± 1.36	98.71 ± 1.50	0.01 ± 0.006	100%	0.082	0.33

**Table 14:** Siena Teacher Model Epileptic Seizure Prediction Results.

Prediction Methods	AUC (%)	Sen (%)	FPR/h	Sig. (%)	Params (M)	Model Size (MB)
CBAM-3D CNN-LSTM [39]	97.32 ± 2.75	97.18 ± 2.44	0.05 ± 0.029	100%	0.622	2.48
Gatformer [7]	97.93 ± 2.52	97.69 ± 3.13	0.04 ± 0.023	100%	0.941	3.76
GAT-TCN [6]	96.29 ± 3.07	95.88 ± 3.58	0.06 ± 0.024	100%	0.812	3.24
Multidimensional Transformer & LSTM-GRU Fusion [8]	97.08 ± 2.47	97.22 ± 3.00	0.04 ± 0.021	100%	1.103	4.40
RGF-Model	98.97 ± 1.51	98.39 ± 2.01	0.02 ± 0.013	100%	0.082	0.33

**Table 15:** CHB-MIT Teacher Model Epilepsy Seizure Detection Results.

Detection Methods	Acc (%)	Sen (%)	Spe (%)	AUC (%)	Params (M)	Model Size (MB)
SDCAE [9]	98.79 ± 0.91	98.72 ± 1.20	98.85 ± 0.90	98.80 ± 0.83	0.415	1.64
CNN-LSTM -SAT [40]	98.91 ± 1.05	98.72 ± 1.54	99.14 ± 1.40	99.07 ± 1.25	0.533	2.12
GTN [41]	98.43 ± 1.24	97.36 ± 2.15	99.23 ± 1.22	98.35 ± 0.84	0.781	3.12
CNN-LSTM -Bilinear [10]	98.84 ± 0.90	98.69 ± 1.10	97.97 ± 1.38	98.56 ± 1.26	0.724	2.88
RGF-Model	98.78 ± 1.18	98.36 ± 1.46	99.12 ± 0.67	98.72 ± 1.17	0.082	0.33

**Table 16:** Siena Teacher Model Epilepsy Seizure Detection Results.

Detection Methods	Acc (%)	Sen (%)	Spe (%)	AUC (%)	Params (M)	Model Size (MB)
SDCAE [9]	97.89 ± 1.80	98.12 ± 1.20	98.17 ± 1.35	98.21 ± 1.42	0.415	1.64
CNN-LSTM -SAT [40]	98.15 ± 1.29	98.11 ± 1.46	98.23 ± 1.93	98.31 ± 1.28	0.533	2.12
GTN [41]	97.48 ± 2.00	97.36 ± 2.07	97.53 ± 2.71	97.65 ± 2.07	0.781	3.12
CNN-LSTM -Bilinear [10]	97.98 ± 1.44	98.14 ± 1.67	97.97 ± 1.59	98.25 ± 1.88	0.724	2.88
RGF-Model	98.92 ± 1.69	98.54 ± 1.49	99.11 ± 1.57	98.96 ± 1.77	0.082	0.33

**Table 17:** Comparison of CHB-MIT and Siena datasets with existing KD models for seizure prediction.

Dataset	Methods	AUC (%)	Sen (%)	FPR/h	Sig. (%)	Params (M)	Model Size (MB)
CHB-MIT	Cross-Subject KD [19]	90.21 ± 3.86	95.25 ± 2.94	0.11 ± 0.022	100%	0.537	2.03
	KDTT [51]	85.36 ± 3.89	90.74 ± 3.87	0.1 ± 0.028	100%	2.221	8.85
	MoKD [18]	88.17 ± 3.82	93.43 ± 2.62	0.12 ± 0.024	100%	2.214	8.74
	RGF-Model	99.54 ± 1.36	98.71 ± 1.50	0.01 ± 0.006	100%	0.082	0.33
Siena	Cross-Subject KD [19]	89.91 ± 3.61	90.07 ± 3.67	0.17 ± 0.035	100%	0.537	2.03
	KDTT [51]	84.26 ± 4.55	85.77 ± 3.67	0.23 ± 0.045	87.50%	2.221	8.85
	MoKD [18]	87.59 ± 4.28	88.17 ± 3.46	0.22 ± 0.040	75.00%	2.214	8.74
	RGF-Model	98.97 ± 1.51	98.39 ± 2.01	0.02 ± 0.013	100%	0.082	0.33

**Table 18:** Comparison of CHB-MIT and Siena datasets with existing KD models for seizure detection.

Dataset	Methods	Acc (%)	Sen (%)	Spe (%)	AUC (%)	Params (M)	Model Size (MB)
CHB-MIT	M2SKD [18]	93.56 ± 2.33	92.51 ± 1.68	94.26 ± 1.90	95.74 ± 1.75	2.252	8.77
	KD-Channel- Pruning [52]	91.05 ± 2.82	90.41 ± 2.89	91.74 ± 2.56	92.52 ± 1.97	0.003	0.01
	KDTL [53]	94.62 ± 1.88	93.35 ± 1.80	95.40 ± 1.58	94.71 ± 2.17	4.235	16.85
	RGF-Model	98.78 ± 1.18	98.36 ± 1.46	99.12 ± 0.67	98.72 ± 1.17	0.082	0.33
Siena	M2SKD [18]	92.31 ± 1.81	91.97 ± 3.23	92.58 ± 2.42	92.22 ± 2.48	2.252	8.77
	KD-Channel- Pruning [52]	90.87 ± 3.07	90.11 ± 3.43	90.24 ± 3.04	90.15 ± 3.47	0.003	0.01
	KDTL [53]	93.84 ± 2.20	93.16 ± 2.36	93.88 ± 1.97	93.57 ± 2.02	4.235	16.85
	RGF-Model	98.92 ± 1.69	98.54 ± 1.49	99.11 ± 1.57	98.96 ± 1.77	0.082	0.33

**Table 19:** On-device efficiency benchmarks under a standardized CPU baseline.

	Role	Methods	Params (M)	Model Size (MB)	FLOPs per 2-s Window (M)	Peak RAM (MB)	Latency per 2-s window (ms) ± SD	Real- Time Factor	Energy per Inference (mJ, Estimated)
Prediction	student	RGF-Model	0.082	0.33	35	70	11.8 ± 0.9	0.0059	1.4
	Teacher A	Multidimensional Transformer & LSTM-GRU Fusion [8]	1.103	4.40	210	180	28.6 ± 2.0	0.0143	8.4
	Teacher B	Gatformer [7]	0.941	3.76	240	200	32.4 ± 2.3	0.0162	9.6
	Representative SOTA	CBAM-3D CNN-LSTM [39]	0.622	2.48	320	220	44.8 ± 3.1	0.0224	12.8
	Representative SOTA	GAT-TCN [6]	0.812	3.24	260	190	36.9 ± 2.6	0.0185	10.4
Detection	student	RGF-Model	0.082	0.33	35	70	11.7 ± 0.8	0.0058	1.4
	Teacher A	CNN-LSTM-SAT [40]	0.533	2.12	190	170	26.1 ± 1.8	0.0131	7.6
	Teacher B	SDCAE [9]	0.415	1.64	160	150	22.4 ± 1.6	0.0112	6.4
	Representative SOTA	GTN [41]	0.781	3.12	380	230	53.6 ± 3.7	0.0268	15.2
	Representative SOTA	CNN-LSTM -Bilinear [10]	0.724	2.88	300	210	41.3 ± 2.9	0.0207	12.0

## Data Availability

The CHB-MIT dataset and the Siena dataset used in this study are open datasets. It can be used for research purposes and is open to all, subject to specific terms. The CHB-MIT dataset is specified and the open access link is: https://physionet.org/content/chbmit/1.0.0/, accessed on 15 December 2022. The Siena dataset follows (CC BY-NC-ND 4.0) and is specified and the open access link is: https://physionet.org/content/siena-scalp-eeg/1.0.0/, accessed on 14 September 2023.
